# Patterns of global burden of 13 diseases attributable to lead exposure, 1990–2019

**DOI:** 10.1186/s12889-023-15874-7

**Published:** 2023-06-12

**Authors:** Tongtong Xu, Kangqian Lin, Miao Cao, Xinlu Miao, Heng Guo, Dongsheng Rui, Yunhua Hu, Yizhong Yan

**Affiliations:** 1 Department of Preventive Medicine, School of Medicine, Shihezi University, No. 59, North 2nd Rd, Hong-Shan District, Shihezi, 832003 Xinjiang China; 2 Key Laboratory of Preventive Medicine, Shihezi University, Shihezi, Xinjiang China; 3 Key Laboratory of Xinjiang Endemic and Ethnic Diseases (Ministry of Education), School of Medicine, Shihezi University, Shihezi, Xinjiang China; 4 Key Laboratory for Prevention and Control of Crucial Emerging Infectious Diseases and Public, Health Security of The Xinjiang Production and Construction Corps, Shihezi, Xinjiang China

**Keywords:** Global burden, Lead exposure, Attribution analysis, Epidemiology

## Abstract

**Objectives:**

Understanding the spatio-temporal patterns of the global burden of various diseases resulting from lead exposure is critical for controlling lead pollution and disease prevention.

**Methods:**

Based on the 2019 Global Burden of Disease (GBD) framework and methodology, the global, regional, and national burden of 13 level-three diseases attributable to lead exposure were analyzed by disease type, patient age and sex, and year of occurrence. Population attributable fraction (PAF), deaths and disability-adjusted life years (DALYs), age-standardized mortality rate (ASMR) and age-standardized DALYs rate (ASDR) obtained from the GBD 2019 database were used as descriptive indicators, and the average annual percentage change (AAPC) was estimated by a log-linear regression model to reflect the time trend.

**Results and conclusions:**

From 1990 to 2019, the number of deaths and DALYs resulting from lead exposure increased by 70.19% and 35.26%, respectively; however, the ASMR and ASDR decreased by 20.66% and 29.23%, respectively. Ischemic heart disease (IHD), stroke, and hypertensive heart disease (HHD) showed the highest increases in deaths; IHD, stroke, and diabetes and kidney disease (DKD) had the fastest-growing DALYs. The fastest decline in ASMR and ASDR was seen in stroke, with AAPCs of -1.25 (95% CI [95% confidence interval]: -1.36, -1.14) and -1.66 (95% CI: -1.76, -1.57), respectively. High PAFs occurred mainly in South Asia, East Asia, the Middle East, and North Africa. Age-specific PAFs of DKD resulting from lead exposure were positively correlated with age, whereas the opposite was true for mental disorders (MD), with the burden of lead-induced MD concentrated in children aged 0–6 years. The AAPCs of ASMR and ASDR showed a strong negative correlation with the socio-demographic index. Our findings showed that the global impact of lead exposure and its burden increased from 1990 to 2019 and varied significantly according to age, sex, region, and resulting disease. Effective public health measures and policies should be adopted to prevent and control lead exposure.

**Supplementary Information:**

The online version contains supplementary material available at 10.1186/s12889-023-15874-7.

## Introduction

Lead is a toxic heavy metal that is recognized as a hazardous environmental pollutant by the World Health Organization [1]. However, it is frequently used in various products, such as gasoline, pigments, ceramic glazes, and lead-painted toys. Humans may be exposed to lead in various ways, such as through inhalation of lead dust and ingesting lead-containing water and food [1, 2]. Dietary intake of lead has become the main pathway for humans, especially at low levels of lead exposure [3]. As reported, 68% of the 268 US Total Diet Study (TDS) food samples had detectable levels of lead [4], while lead residues were detected in 99.3% of samples in the Chinese TDS [5]. Moreover, as human organs and tissues have no effective mechanism to excrete lead, its concentration inside the body accumulates and increases with age [6]. Although the early recognition of the hazards of lead and conscious efforts to reduce lead utilization have led to a decrease in lead use in developed regions [7], exposure levels in many other regions remain rather high [8]. It was estimated that approximately half of the two million deaths resulting from exposure to known chemicals in 2019 were due to lead exposure, and that millions of people, many of them children, were exposed to low levels of lead. Therefore, lead represents a major global environmental hazard and poses a major threat to human health [9, 10].

Lead exposure at any level is unsafe. A previous study confirmed that lead can negatively affect human health even at low levels because long-term exposure to lead has a cumulative effect [11], and affects organs differently, being associated with heart failure [12], inhibition of hepatic gluconeogenesis [13], ovarian damage [14], and nephrotoxicity [15]. Moreover, it can spread between organs and tissues, causing accumulation in the intestines, lungs, liver, spleen, kidneys, central nervous system and bones, thereby interfering with a variety of physiological processes [16]. Previous studies have shown that lead affects the function of many systems, organs, and tissues, including the nervous system [17], cardiovascular system [18], immune system [19], reproductive system [20], kidney [21] and bone tissue [22]. Additionally, maternal prenatal lead exposure puts the developing fetus at risk of neurodevelopmental defects owing to lead crossing the placental barrier [23], and exposure during the later childhood has a negative intellectual impact, mainly due to lead damaging the nervous system [24]. Moreover, exposure during childhood has long-lasting effects [25]. Although lead exposure can also cause cognitive delays in adults [26], it mainly results in increased blood pressure [27] and diabetes [28]. This highlights that lead exposure can cause various health problems and lead to multiple diseases in different populations, making it a global health threat, even at low exposure levels.

**Table 1:** Global deaths attributable to lead exposure in 1990 and 2019, and the temporal trend from 1990 to 2019

Cause of deaths	1990					2019				1990–2019		
	Deaths No.×10^3^ (95%UI)	ASMR per 100 000 (95%UI)	Age-standardized PAF, % (95%UI)			Deaths No.×10^3^ (95% UI))	ASMR per 100 000 (95%UI)	Age-standardized PAF, % (95%UI)		AAPC of ASMR (95%CI)	AAPC of age-standardized PAF (95%CI)	
**All causes**												
Both	529.84 (312.63, 772.03)	14.47 (8.40, 21.43)		1.30 (0.76, 1.93)		901.72 (550.91, 1288.85)	11.48 (7.00,16.49)	1.56 (0.95, 2.23)		-0.81 (-0.85, -0.77)	0.67 (0.62, 0.72)	
Female	196.71 (99.62, 309.96)	9.83 (4.87, 15.66)		1.03 (0.51, 1.64)		345.77 (188.45, 532.92)	7.88 (4.29,12.15)	1.28 (0.70, 1.96)		-0.76 (-0.87, -0.64)	0.76 (0.68, 0.83)	
Male	333.13 (210.41, 463.22)	20.52 (12.84, 28.84)		1.57 (0.99, 2.22)		555.95 (357.23, 766.84)	16.05 (10.26, 21.96)	1.84 (1.18, 2.52)		-0.85 (-0.94, -0.75)	0.53 (0.42, 0.64)	
**Disease type**												
**Cardiovascular diseases**	508.45 (299.71, 745.95)	13.89 (7.99, 20.55)		3.92 (2.28, 5.72)		848.78 (517.04, 1212.10)	10.80 (6.56, 15.50)	4.50 (2.77, 6.41)		-0.88 (-0.92, -0.83)	0.48 (0.41, 0.56)	
Rheumatic heart disease	9.39 (5.10, 16.10)	0.23 (0.12, 0.41)		2.55 (1.44, 4.40)		7.69 (4.09, 13.50)	0.10 (0.05, 0.17)	2.48 (1.32, 4.56)		-2.98 (-3.09, -2.88)	-0.10 (-0.21, 0.02)	
Ischemic heart disease	220.99 (124.89, 334.74)	6.12 (3.36, 9.46)		3.59 (2.00, 5.51)		413.04 (242.84, 615.75)	5.26 (3.08, 7.88)	4.46 (2.67, 6.58)		-0.54 (-0.59, -0.48)	0.74 (0.60, 0.88)	
Stroke	207.41 (124.08, 304.80)	5.53 (3.28, 8.22)		4.18 (2.52, 6.04)		305.27 (182.80, 435.67)	3.85 (2.30, 5.50)	4.57 (2.81, 6.56)		-1.25 (-1.36, -1.14)	0.31 (0.23, 0.39)	
Hypertensive heart disease	57.00 (22.33, 125.06)	1.61 (0.57, 3.54)		8.32 (3.04, 18.07)		97.49 (30.51, 225.24)	1.27 (0.39, 2.96)	8.38 (2.64, 18.73)		-0.82 (-0.97, -0.67)	0.03 (-0.02, 0.07)	
Non-rheumatic valvular heart disease	0.94 (0.41, 1.59)	0.03 (0.01, 0.05)		1.13 (0.49, 1.97)		1.92 (0.83, 3.40)	0.03 (0.01, 0.05)	1.15 (0.50, 2.06)		-0.29 (-0.49, -0.10)	0.05 (< 0.01, 0.10)	
Cardiomyopathy and myocarditis	2.56 (1.05, 4.52)	0.08 (0.03, 0.14)		1.09 (0.45, 1.97)		3.48 (1.45, 6.35)	0.04 (0.02, 0.08)	1.02 (0.43, 1.83)		-1.77 (-1.82, -1.71)	-0.22 (-0.58, 0.16)	
Atrial fibrillation and flutter	2.35 (1.21, 3.77)	0.08 (0.04, 0.13)		1.82 (0.93, 2.91)		7.10 (4.00, 11.09)	0.10 (0.05, 0.15)	2.20 (1.24, 3.34)		0.72 (0.66, 0.78)	0.64 (0.60, 0.69)	
Aortic aneurysm	1.99 (0.90, 3.39)	0.05 (0.02, 0.09)		1.97 (0.92, 3.19)		3.47 (1.74, 5.61)	0.04 (0.02, 0.07)	1.97 (1.02, 3.12)		-0.70 (-0.78, -0.62)	0.02 (-0.03, 0.06)	
Peripheral artery disease	0.30 (0.10, 0.68)	0.01 (0.00, 0.02)		0.97 (0.36, 1.79)		0.79 (0.30, 1.68)	0.01 (< 0.01, 0.02)	1.07 (0.43, 1.95)		0.24 (0.20, 0.29)	0.36 (0.30, 0.43)	
Endocarditis	0.61 (0.31, 1.02)	0.02 (0.01, 0.03)		2.13 (1.11, 3.41)		1.19 (0.55, 2.09)	0.02 (0.01, 0.03)	1.75 (0.81, 3.03)		-0.06 (-0.14, 0.02)	-0.67 (-0.71, -0.62)	
Other cardiovascular and circulatory diseases	4.90 (2.70, 7.84)	0.13 (0.07, 0.21)		2.46 (1.38, 3.75)		7.34 (4.30, 10.81)	0.09 (0.05, 0.14)	2.57 (1.56, 3.77)		-1.18 (-1.24, -1.13)	0.16 (0.11, 0.20)	
**Diabetes and kidney diseases**	21.39 (12.89, 30.89)	0.59 (0.35, 0.85)		1.70 (1.03, 2.46)		52.94 (31.64, 76.23)	0.68 (0.40, 0.98)	1.79 (1.10, 2.56)		0.50 (0.39, 0.60)	0.15 (0.08, 0.23)	
Chronic kidney disease	21.39 (12.89, 30.89)	0.59 (0.35, 0.85)		3.63 (2.20, 5.22)		52.94 (31.64, 76.23)	0.68 (0.40, 0.98)	3.70 (2.28, 5.29)		0.50 (0.39, 0.60)	0.06 (-0.03, 0.14)	

Note: ASMR, age-standardized mortality rate; PAF, population attributable fraction; AAPC, average annual percentage change; UI, uncertainty interval; CI, confidence interval

Strong epidemiological evidence, such as a comprehensive quantitative assessment of the disease burden attributable to lead exposure, would aid the development of effective prevention strategies that reduce the hazards of lead exposure. However, to date, there has been no comprehensive, accurate description of the global disease burden of lead exposure. Therefore, this study comparatively assessed the burden and trends of multiple diseases attributable to lead exposure among different populations at global, regional, and national levels using the latest data from the Global Burden of Disease Study (GBD), aiming to provide a comprehensive basis for the scientific, precise development of lead exposure prevention and control strategies.

## Materials and methods

### Data collection

Data on the burden of diseases attributable to lead exposure were obtained from the 2019 Global Burden of Disease Study (GBD 2019), available for download through the Institute for Health Metrics and Evaluation (IHME, http://ghdx.healthdata.org/gbdresults-tool gbd-results-tool). The database provides a comprehensive estimation and integrated evaluation of 369 diseases and 87 risk factors in 204 countries worldwide and is organized by country, year of occurrence, sex and age. Detailed methods for data collection, processing, and modeling have been described in previous studies [29, 30].

The socio-demographic index (SDI) is a novel development classification indicator that is closely associated with social development and population health outcomes, with values ranging from 0 to 1, where a higher value indicates a higher level of development related to health outcomes [29]. The 204 countries are classified into five SDI categories based on lag-distributed income per capita, fertility rates among women under the age of 25, and mean education for individuals aged 15 years and older: low SDI (≤ 0.454743), low-middle SDI (0.454743–0.607679), middle SDI (0.607679–0.689504), high-middle SDI (0.689504–0.805129), and high SDI (> 0.805129) levels, respectively [31]. Additionally, the GBD divides these countries into 21 regions and seven super-regions based on geographic location [29].

In this study, GBD data on the global burden of the following 13 diseases caused by lead exposure were used: cardiovascular diseases (CVD) (aortic aneurysm, atrial fibrillation and flutter, cardiomyopathy and myocarditis, endocarditis, hypertensive heart disease [HHD], ischemic heart disease [IHD], non-rheumatic valvular heart disease, other cardiovascular and circulatory diseases, peripheral artery disease, rheumatic heart disease [RHD], and stroke), diabetes and kidney disease (DKD), and mental disorders (MD). Regional and patient age and sex differences affecting the burden of these diseases and their trends over time were analyzed and compared.

### Data analysis

The number of deaths and disability-adjusted life years (DALYs), mortality rate, DALY rate, age-standardized mortality rate (ASMR), age-standardized DALY rate (ASDR), population attribution fraction (PAF), and their 95% uncertainty intervals (95% UI) (interquartile range based on values ranked 25th and 975 out of a random sample of 1000) were used to analyze the global burden of the selected diseases from 1990 to 2019. All the above indicators were from GBD 2019, and the calculation method has been reported in previous studies [29, 32].

Jointpoint regression model is a set of linear statistical models widely used to assess the trend of disease burden over time. It includes a linear regression model (*y = xb*) and a log-linear regression model (*ln y = xb*), the latter of which is commonly used to analyze population-based mortality trends [33]. Therefore, a log-linear regression model was adopted in this study.

**Table 2:** Global DALYs attributable to lead exposure in 1990 and 2019, and the temporal trend from 1990 to 2019

Cause of DALYs	1990				2019				1990–2019	
	DALYs No.×10^5^ (95% UI)	ASDR per 100 000 (95%UI)	Age-standardized PAF, % No. (95%UI)		DALYs No.×10^5^ (95% UI)	ASDR per 100 000 (95%UI)	Age-standardized PAF, % No. (95%UI)		AAPC of ASDR (95%CI)	AAPC of age-standardized PAF (95%CI)
**All causes**										
Both	160.25 (103.21, 221.68)	378.01 (240.55, 524.18)	0.76 (0.48, 1.05)		216.76 (138.13, 302.98)	267.52 (170.57, 373.44)	0.82 (0.53, 1.14)		-1.19 (-1.24, -1.14)	0.28 (0.23, 0.33)
Female	58.31 (34.51, 84.98)	260.52 (150.50, 383.12)	0.56 (0.33, 0.82)		80.43 (48.18, 117.28)	188.85 (113.07, 274.64)	0.62 (0.38, 0.90)		-1.12 (-1.19, -1.05)	0.36 (0.29, 0.44)
Male	101.94 (68.25, 136.87)	512.87 (339.34, 692.87)	0.95 (0.63, 1.28)		136.34 (88.81, 188.24)	357.56 (233.38, 493.30)	1.00 (0.67, 1.37)		-1.24 (-1.30, -1.18)	0.20 (0.15, 0.26)
**Disease type**										
**Cardiovascular diseases**	128.85 (77.34, 186.47)	318.54 (190.85, 461.21)	4.50 (2.70, 6.45)		177.35 (104.88, 256.6)	216.80 (128.35, 314.82)	4.46 (2.67, 6.35)		-1.32 (-1.39, -1.25)	-0.04 (-0.16, 0.08)
Rheumatic heart disease	3.20 (1.69, 5.44)	7.18 (3.87, 12.16)	2.53 (1.40, 4.25)		2.14 (1.07, 3.73)	2.58 (1.28, 4.55)	1.95 (1.00, 3.42)		-3.46 (-3.58, -3.34)	-0.91 (-1.02, -0.80)
Ischemic heart disease	54.09 (31.78, 79.51)	134.35 (78.06, 199.09)	4.27 (2.50, 6.29)		83.69 (48.96, 124.49)	102.26 (59.74, 152.87)	4.56 (2.72, 6.68)		-0.94 (-1.01, -0.88)	0.22 (0.04, 0.40)
Stroke	54.37 (32.53, 79.52)	133.36 (80.09, 195.09)	4.88 (2.94, 7.03)		67.39 (39.12, 98.16)	81.97 (47.86, 119.11)	4.64 (2.73, 6.69)		-1.66 (-1.76, -1.57)	-0.18 (-0.26, -0.09)
Hypertensive heart disease	13.08 (5.90, 26.76)	33.36 (14.33, 69.08)	9.11 (4.15, 18.20)		17.70 (6.64, 38.45)	22.04 (8.09, 47.48)	8.20 (3.12, 17.26)		-1.43 (-1.56, -1.30)	-0.36 (-0.42, -0.31)
Non-rheumatic valvular heart disease	0.20 (0.09, 0.35)	0.53 (0.23, 0.88)	1.18 (0.54, 1.95)		0.32 (0.15, 0.54)	0.40 (0.18, 0.68)	1.12 (0.52, 1.89)		-0.92 (-1.09, -0.75)	-0.19 (-0.25, -0.12)
Cardiomyopathy and myocarditis	0.62 (0.26, 1.08)	1.57 (0.65, 2.70)	0.97 (0.41, 1.68)		0.74 (0.29, 1.37)	0.92 (0.36, 1.68)	0.80 (0.32, 1.47)		-1.85 (-1.88, -1.81)	-0.69 (-0.92, -0.46)
Atrial fibrillation and flutter	1.02 (0.53, 1.65)	2.75 (1.44, 4.45)	2.50 (1.40, 3.71)		2.27 (1.25, 3.63)	2.85 (1.56, 4.57)	2.66 (1.55, 3.89)		0.13 (0.10, 0.15)	0.22 (0.19, 0.25)
Aortic aneurysm	0.47 (0.21, 0.81)	1.15 (0.51, 2.00)	2.26 (1.06, 3.66)		0.70 (0.35, 1.15)	0.85 (0.42, 1.40)	2.08 (1.05, 3.34)		-1.06 (-1.19, -0.93)	-0.29 (-0.40, -0.19)
Peripheral artery disease	0.10 (0.04, 0.19)	0.27 (0.11, 0.54)	1.22 (0.55, 2.08)		0.20 (0.09, 0.38)	0.25 (0.11, 0.48)	1.30 (0.60, 2.20)		-0.26 (-0.30, -0.22)	0.23 (0.19, 0.27)
Endocarditis	0.20 (0.10, 0.33)	0.45 (0.22, 0.74)	1.97 (0.99, 3.27)		0.28 (0.13, 0.50)	0.34 (0.15, 0.62)	1.55 (0.71, 2.69)		-0.92 (-1.07, -0.77)	-0.80 (-0.84, -0.76)
Other cardiovascular and circulatory diseases	1.50 (0.80, 2.43)	3.56 (1.94, 5.75)	2.34 (1.28, 3.67)		1.92 (1.04, 3.04)	2.33 (1.26, 3.69)	2.10 (1.16, 3.26)		-1.45 (-1.50, -1.40)	-0.36 (-0.46, -0.27)
**Mental disorders**	25.18 (11.43, 43.54)	44.18 (19.98, 76.61)	2.80 (1.31, 4.70)		27.16 (12.10, 48.35)	35.70 (15.89, 63.6)	2.29 (1.06, 3.89)		-0.74 (-0.75, -0.72)	-0.70 (-0.71, -0.69)
Idiopathic developmental intellectual disability	25.18 (11.43, 43.54)	44.18 (19.98, 76.61)	66.50 (35.59, 84.36)		27.16 (12.1, 48.35)	35.70 (15.89, 63.6)	61.94 (32.07, 80.16)		-0.74 (-0.75, -0.72)	-0.25 (-0.25, -0.24)
**Diabetes and kidney diseases**	6.23 (3.73, 9.08)	15.29 (9.19, 22.14)	1.29 (0.76, 1.88)		12.25 (7.08, 18.18)	15.02 (8.68, 22.26)	1.09 (0.62, 1.64)		-0.06 (-0.17, 0.04)	-0.57 (-0.67, -0.47)
Chronic kidney disease	6.23 (3.73, 9.08)	15.29 (9.19, 22.14)	3.16 (1.90, 4.63)		12.25 (7.08, 18.18)	15.02 (8.68, 22.26)	2.92 (1.70, 4.33)		-0.06 (-0.17, 0.04)	-0.28 (-0.36, -0.21)

Note: DALYs, disability-adjusted life years; ASDR, age-standardized DALY rate; PAF, population attributable fraction; AAPC, average annual percentage change; UI, uncertainty interval; CI, confidence interval

In this model, the least square method is used to calculate the sum of squares of the residual difference between the estimated value and the actual value, and the inflection point of the motion trend is obtained, which is then divided into several segments. The regression coefficients of each segment were then weighted to obtain the average annual percentage change (AAPC) to estimate the overall change over the study period [34, 35], as follows:

$$ {ln}(\gamma )=\alpha +{\beta }_{i}\chi +\epsilon $$

$$ APCs=100\times \left({exp}({\beta }_{i}\right)-1)$$

$$ AAPCs=\left\{{exp}(\sum {\omega }_{i}{\beta }_{i}/\sum {\omega }_{i})-1\right\}\times 100$$

where $\gamma$ is the respective age-standardized indicators, $\chi$ the calendar year, $\alpha$ the intercept, and $\epsilon$ the error. ${\beta }_{i}$ is expressed as the slope coefficients of each segment in the expected year range; then ${\omega }_{i}$ is the year length of each segment in the year range.

Age-standardized indicators were considered to be increasing if the smallest 95% CI of the corresponding AAPC estimate was > 0, decreasing if the largest 95% CI was < 0, and stable if the 95% CI = 0. Furthermore, to explore the impact of the SDI on the burden of diseases resulting from lead exposure, associations were assessed at the national and regional level using a scatter plot and Spearman correlation analysis, considering the non-normal distribution of corresponding variables.

All statistical analyses were performed using R version 4.2.1 (https://www.r-project.org/), and the two-sided P value of less than 0.05 was considered statistically significant.

## Results

### PAFs of diseases attributable to lead exposure

Global age-standardized death-PAF attributable to lead exposure increased from 1.30% (95% UI: 0.76%, 1.93%) in 1990 to 1.56% (95% UI: 0.95%, 2.23%) in 2019, with an AAPC of 0.67 (95% CI: 0.62, 0.72) (Table 1). DALY-PAF increased from 0.76% (95% UI: 0.48%, 1.05%) in 1990 to 0.82% (95% UI: 0.53%, 1.14%) in 2019, with an AAPC of 0.28 (95% CI: 0.23, 0.33) (Table 2).

**Fig. 1:**
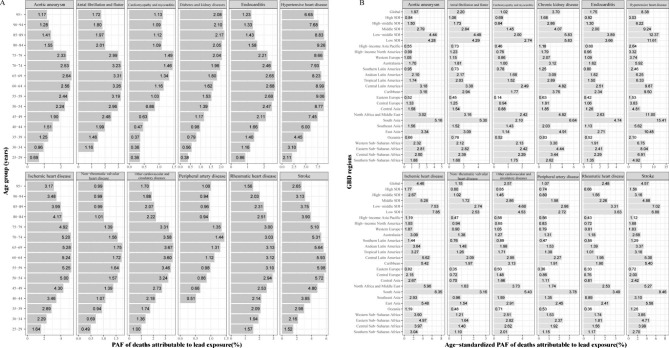
PAF of specific GBD level-three diseases in ASMR attributable to lead exposure by age group and by region for both sexes in 2019. (**A**) By age group. (**B**) By region. ASMR, age-standardized mortality rate; GBD, Global Burden of Disease Study; SDI, socio-demographic index

The patterns of death-PAFs attributable to lead exposure at different ages varied considerably between the 13 included level-three diseases. The age-specific PAF of deaths from DKD showed a significantly positive correlation with age, while that for HHD showed a bimodal distribution, with peaks occurring around 55–59 and 80–84 years, and the death-PAFs for the remaining were unimodal with age, peaking at 60–74 years (Fig. 1A; Table S1). A similar pattern was observed in DALY-PAFs, except that MD declined with age. The DALY-PAF in 5 years of MD (25.14%) was 10 to 114 times higher than that in 20–24 years (2.51%) to 95 + years (0.22%) (Fig. S1A; Table S2–S3).

**Fig. 2:**
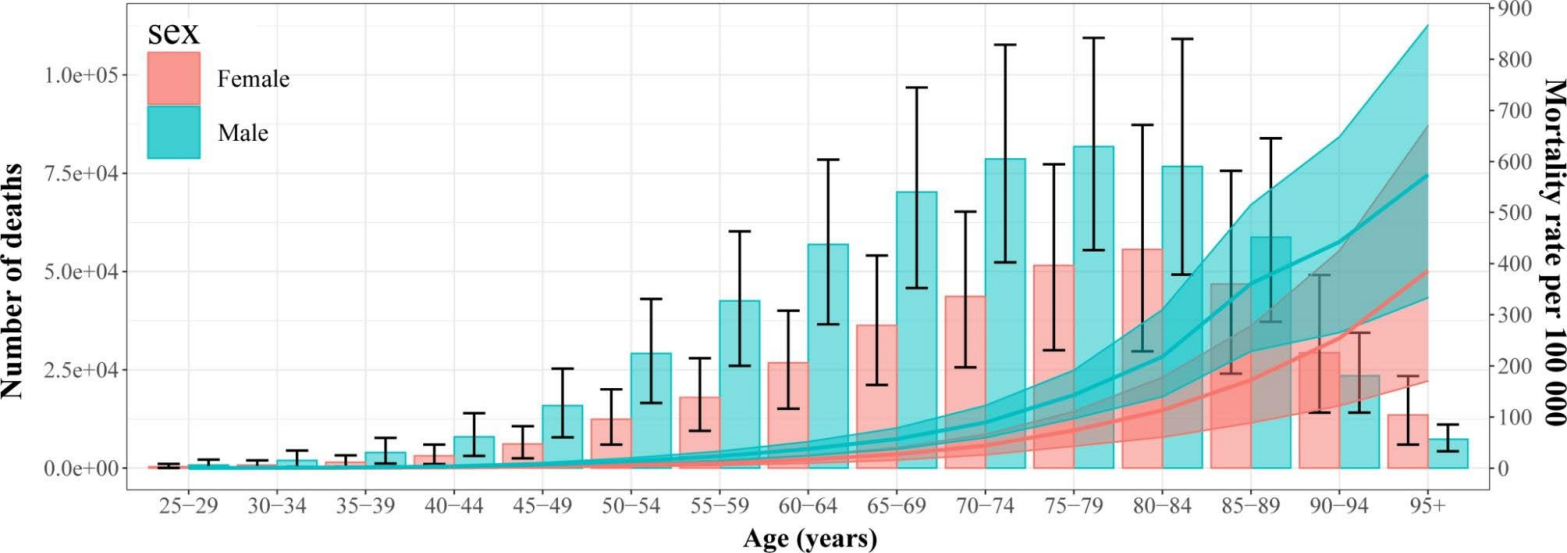
Age-specific numbers (bar plot) and rates (line plot) of deaths attributable to lead exposure in 2019 by sex

The ASMR-PAFs varied between regions, with the highest PAFs mainly occurring in low and low-middle SDI regions, and the lowest in high SDI regions. The highest PAF was found in South Asia and the lowest in Eastern Europe (Fig. 1B). Similar patterns were observed for the ASDR-PAFs (Fig. S1B).

**Fig. 3:**
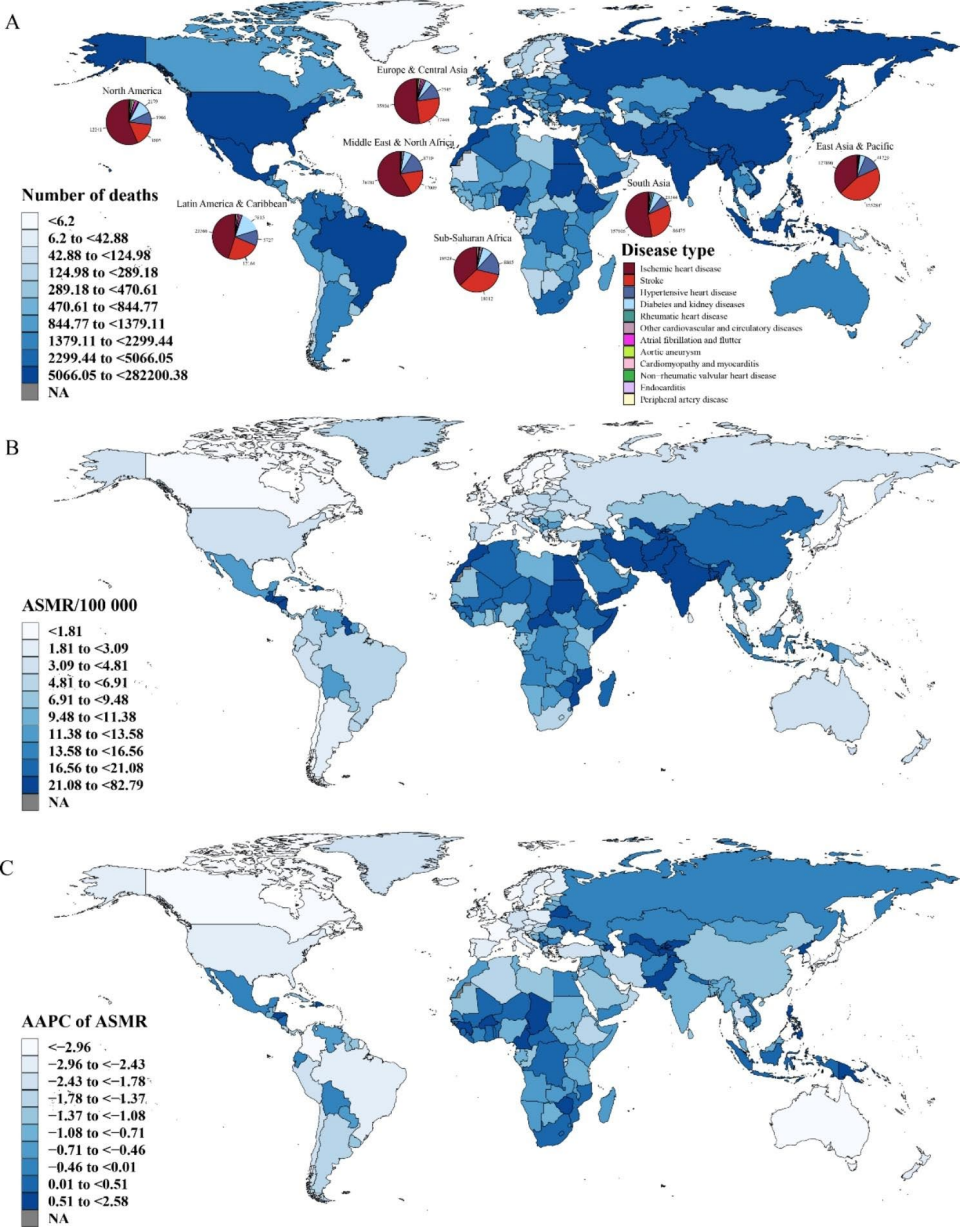
Global mortality burden attributable to lead exposure for both sexes. (**A**) Number of deaths in 2019, and pie plots depict the proportion of specific GBD level-three diseases by World Bank Region (the number represents the number of specific cause). (**B**) ASMR in 2019. (**C**) AAPC of ASMR from 1990 to 2019. ASMR, age-standardized mortality rate; AAPC, average annual percentage change; GBD, Global Burden of Disease Study

Among the 13 diseases, HHD had the highest PAF, followed by stroke and IHD (Tables 1 and 2). From 1990 to 2019, death-PAFs increased for all diseases except for RHD, cardiomyopathy and myocarditis, and endocarditis, with that for IHD increasing the fastest and that for endocarditis declining fastest. The AAPCs were 0.74 (95% CI: 0.60, 0.88) and -0.67 (95% CI: -0.71, -0.62), respectively (Table 1). The DALY-PAFs of all diseases decreased, except for that of IHD, atrial fibrillation and flutter, and peripheral artery disease, with the fastest increase in peripheral artery disease and the fastest decrease in RHD. The AAPCs were 0.23 (95% CI: 0.19, 0.27) and -0.91 (95% CI: -1.02, -0.80), respectively (Table 2).

### Global burden of diseases attributable to lead exposure in 2019

Globally, the number of deaths attributable to lead exposure was 901,720 (95% UI: 550,910, 1,288,850), with 83.99% in people aged ≥ 60 years (757,340), and the ASMR was 11.48 per 100,000 (95% UI: 7.00, 16.49) (Table 1). The DALY attributable to lead exposure was 21.68 million (95% UI: 13.81, 30.30), with 61.21% in people aged ≥ 60 years (13.27 million), and the ASDR was 267.52 per 100,000 (95% UI: 170.57, 373.44) (Table 2).

The global number of deaths in 2019 showed a unimodal distribution with age, peaking at age 75–79 years for males and 80–84 years for females, and the mortality rate in males was higher than that in females before the age of 85–89, after which the trend reversed. Age-specific mortality rates tended to increase with age in both males and females (Fig. 2; Table S4). The pattern of DALYs only differed slightly from those of deaths; the peak occurred at age 65–69 for both males and females, and its trend reversed after the age of 85–89. The age-specific DALY rates in males tended to increase until the age of 85–89, decreasing thereafter, but continually increased in females (Fig. S2; Table S5).

**Fig. 4:**
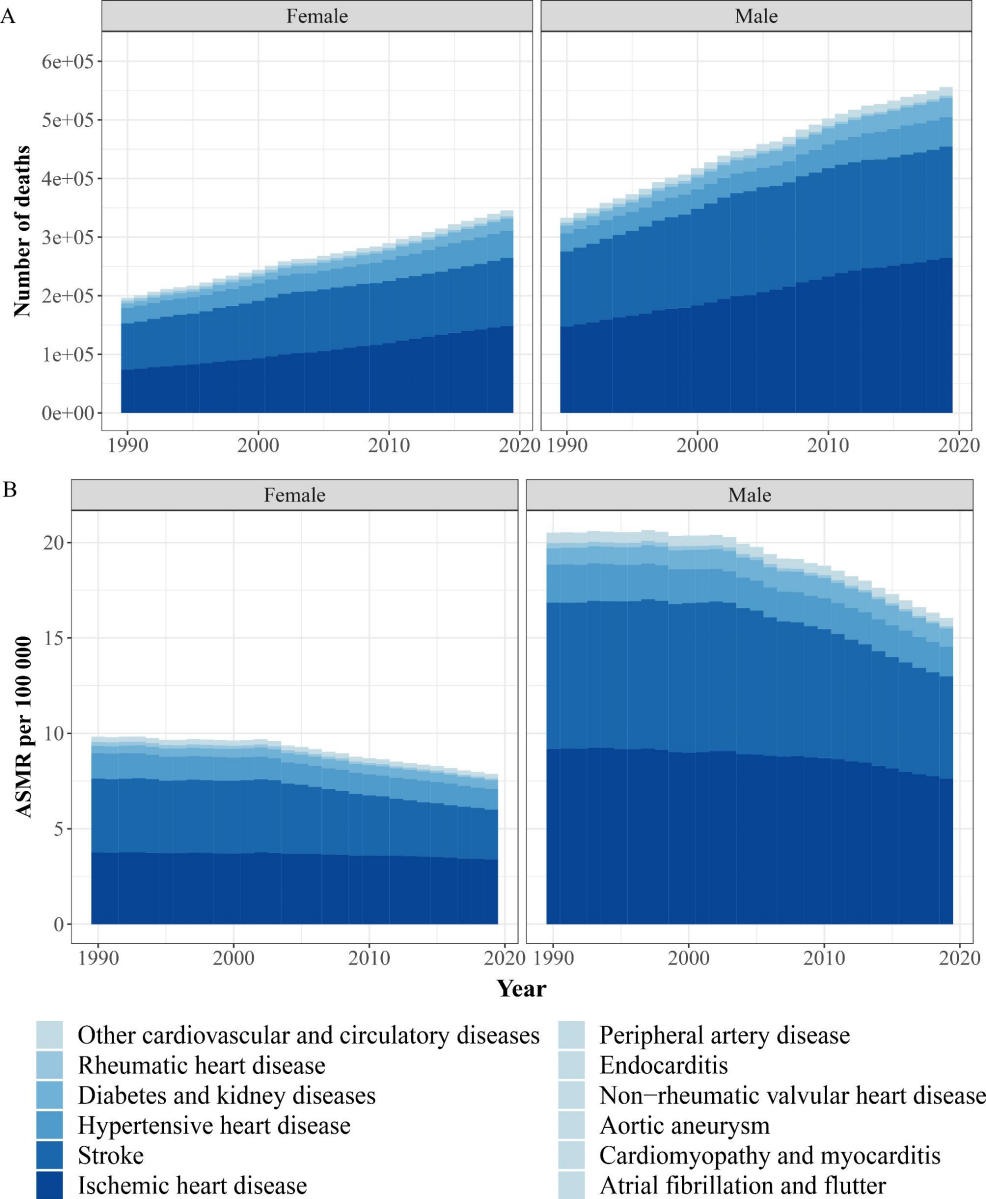
Number of all-age deaths and ASMR attributable lead exposure by sex, 1990–2019. (**A**) Deaths. (**B**) ASMR. ASMR, age-standardized mortality rate

At the SDI region level, the middle SDI region had the highest number of deaths (0.33 million) and DALYs (7.58 million), but the highest ASMR and ASDR occurred in the low-middle and low SDI regions, respectively. Of the 21 GBD regions, the two with the most deaths and DALYs were South Asia and East Asia, while the regions with the highest ASMR and ASDR were South Asia, North Africa, and the Middle East (Table S6–S7). Stroke was found to be the most common cause of death and high DALYs in East Asia and the Pacific; however, IHD was the primary cause in all other regions (Fig. 3A; Fig. S3A).

The three countries with most deaths attributable to lead exposure were China, India, and Bangladesh (Fig. 3A; Table S8), and those with the highest DALYs were India, China and Indonesia (Fig. S3A; Table S9); Bulgaria, Bosnia and Herzegovina, and Malta were the countries with the highest ASMRs (Fig. 3B; Table S8), and Afghanistan, Yemen, and Egypt were those with the highest ASDRs (Fig. S3B; Table S9).

**Fig. 5:**
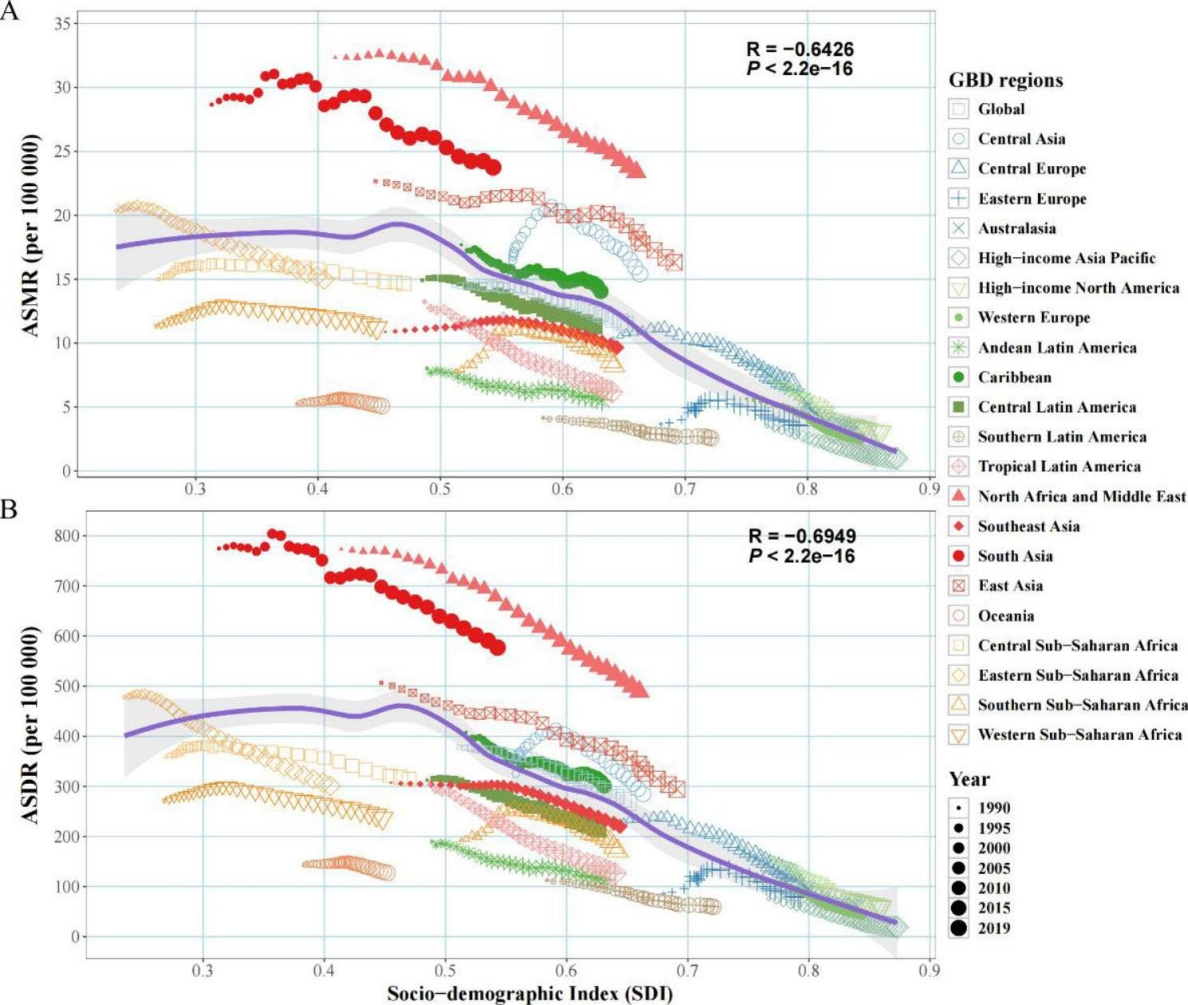
Age-standardized burden rate attributable to lead exposure across 21 GBD regions by socio-demographic index for both sexes, 19902019. (**A**) ASMR; (**B**) ASDR. The purple line was an adaptive association fitted with adaptive Loess regression based on all data points. GBD, Global Burden of Disease Study; ASMR, age-standardized mortality rate; ASDR, age-standardized disability-adjusted life year rate

Out of the 13 level-three diseases, IHD, stroke, and HHD were the three major causes of deaths attributable to lead exposure, accounting for 90.47% of the total, with 413,040 (95% UI: 242.84, 615.75), 305,270 (95% UI: 182.80, 435.67), and 97,490 (95% UI: 30.51, 225.24) deaths, respectively. These three diseases were also those with the highest ASMRs (Table 1). In contrast, the top three diseases according to DALYs and ASDRs were IHD, stroke, and MD (Table 2).

The number of age-specific deaths from each disease showed a unimodal distribution, with peaks occurring at age 70–89. Aortic aneurysm, cardiomyopathy and myocarditis, DKD, other cardiovascular and circulatory diseases, RHD, and stroke peaked at 75–79 years. The age-specific DALY of MD decreased gradually with age, and those of other diseases showed a unimodal distribution, with peaks mainly occurring at the age of 60–74 years. Aortic aneurysm, DKD, endocarditis, IHD, and stroke all peaked at 65–69 years (Fig. S4).

**Fig. 6:**
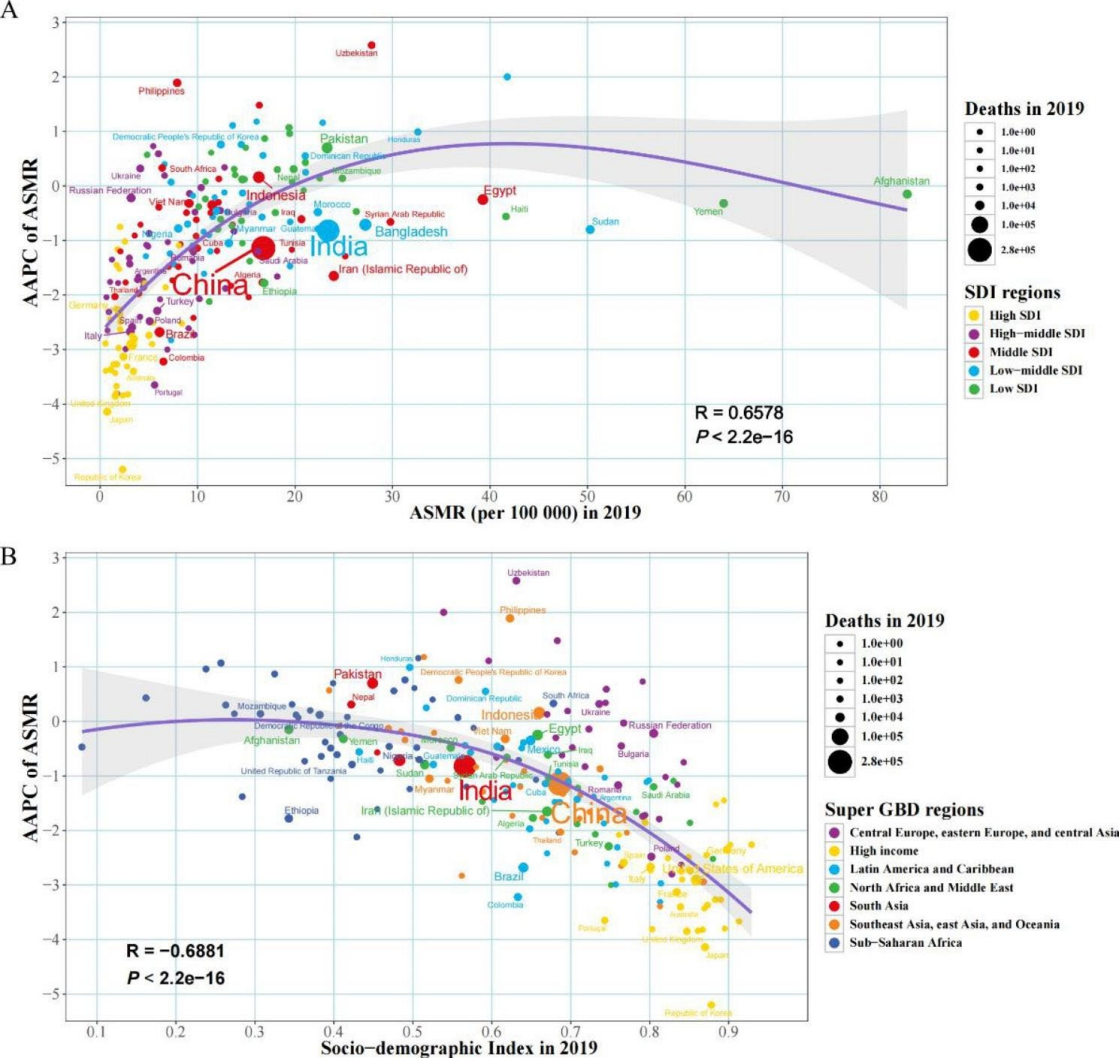
The factors associated with the AAPC of ASMR attributable to lead exposure from 1990 to 2019, both sexes, at the national level. (**A**) The corresponding ASMR in 2019; (**B**) Socio-demographic index in 2019. The purple line was an adaptive association fitted with adaptive Loess regression based on all data points. ASMR, age-standardized mortality rate; AAPC, average annual percentage change; SDI, socio-demographic index; GBD, Global Burden of Disease Study

### Changing patterns of the disease burden attributable to lead exposure from 1990 to 2019

Global deaths attributable to lead exposure increased from 529,840 (95% UI: 312,630, 772,030) in 1990 to 901,720 (95% UI: 550,910, 1,288,850) in 2019, reflecting an increase of 70.19%, while ASMRs decreased from 14.47 to 100,000 (95% UI: 8.40, 21.43) to 11.48 per 100,000 (95% UI: 7.00, 16.49), with an AAPC of -0.81 (95% CI: -0.85, -0.77) (Table 1). Global DALYs increased by 35.25%, from 16.03 million (95% UI: 10.32, 22.17) in 1990 to 21.68 million (95% UI: 13.81, 30.30) in 2019, while ASDRs decreased from 378.01 to 100,000 (95% UI: 240.55, 524.18) to 267.52 per 100,000 (95% UI: 170.57, 373.44), with an AAPC of -1.19 (95% CI: -1.24, -1.14) (Table 2).

From 1990 to 2019, by age group, the total mortality rate and DALY rate for all diseases decreased for people younger than 80 years, but increased for people aged ≥ 80 years. The mortality rate of DKD decreased in people younger than 60 years, remained stable in the 60–69 age group, and increased in the people aged ≥ 70 years. The DALY rates of DKD and MD decreased below 60 years old, remained stable in the 60–64 age group, but increased in the population aged ≥ 65 years. (Table S1–S5).

At the SDI level, ASMRs and ASDRs decreased across all levels, with the fastest decrease in high SDI areas with AAPCs of -3.10 (95% CI: -3.28, -2.91) and -3.29 (95% CI: -3.37, -3.21), respectively. At the GBD level, ASMRs and ASDRs declined in all regions except Central Asia and Sub-Saharan Africa, with fastest declines occurring in the high-income Asia Pacific region, reflected by AAPCs of -4.34 (95% CI: -4.48, -4.20) and -4.69 (95% CI: -4.85, -4.53), respectively (Table S6–S7). Finally, ASMRs and ASDRs declined fastest in Republic of Korea and increased the fastest in Uzbekistan (Fig. 3C; Fig. S3C; Table S8–S10).

For different sexes, the number of deaths for females and males increased from 196,710 to 333,130 in 1990 to 345,770 and 555,950 in 2019, an increase of 75.78% and 66.89% respectively, and the ASMR decreased from 9.83 to 100,000 and 20.52 per 100,000 in 1990 to 7.88 per 100,000 and 16.05 per 100,000 in 2019, with AAPCs of -0.76 (95% CI: -0.87, -0.64) and -0.85 (95% CI: -0.94, -0.75), respectively (Fig. 4; Table 1). The number of DALYs for females and males increased from 5.83 million and 10.19 million in 1990 to 8.04 million and 13.63 million in 2019, an increase of 37.94% and 33.75%, respectively, and the ASDR decreased from 260.52 to 100,000 and 512.87 per 100,000 in 1990 to 188.85 per 100,000 and 357.56 per 100,000 in 2019, with AAPCs of -1.24 (95% CI: -1.30, -1.18) and -1.12 (95% CI: -1.19, -1.05), respectively (Fig. S5; Table 2).

Out of the 13 level-three diseases, IHD, stroke, and HHD were the primary contributors to the increase in global deaths, cumulatively accounting for 88.84% of the overall increase in global deaths from 1990 to 2019. Their ASMRs decreased from 1990 to 2019, with stroke declining the fastest, evidenced by an AAPC of -1.25 (95% CI: -1.36, -1.14) (Table 1; Fig. 4). The major contributions to the overall increase in DALYs were from IHD, stroke, and DKD, cumulatively accounting for 86.07%. The fastest decrease in ASDRs occurred in stroke, with an AAPC of -1.66 (95% CI: -1.76, -1.57) (Table 2; Fig. S5).

### Changing patterns at different SDI levels and baseline burdens

Both ASMRs and ASDRs showed an overall downward trend with increasing SDI and increased slightly when the SDI was less than 0.47 (Fig. 5). AAPCs of ASMRs or ASDRs showed a strong positive correlation compared to the baseline ASMR or ASDR in 2019 (correlation coefficients were around 0.66 and 0.64, respectively), and a strong negative correlation with the SDI in 2019 (correlation coefficients were around -0.69 and -0.68, respectively) (Fig. 6; Fig. S6).

## Discussion

The present study systematically quantified and comparatively assessed the global burden of 13 diseases resulting from lead exposure in different populations as well as their spatio-temporal trends. From 1990 to 2019, the global impact of lead exposure gradually increased, manifested by a gradual increase in PAFs. The number of deaths and DALYs resulting from all diseases except RHD increased significantly, whereas ASMRs and ASDRs decreased. The effects of lead exposure were most significant in low SDI regions, and the resulting disease burden was concentrated in South Asia, North Africa, and the Middle East. The burden of MDs caused by lead exposure was concentrated in children and adolescents, whereas that of CVD and DKD was more pronounced in individuals aged ≥ 60 years. In conclusion, the burden attributable to lead exposure varied considerably with disease, differences in patient age and sex, and across regions.

Children aged 0–6 years and persons aged ≥ 60 years were the most sensitive to lead exposure. We found that lead exposure had the highest DALY-PAFs in children aged 0–6 years, and that this group was more likely to develop MD, which is consistent with a previous study [36] and may be related to the fact that lead reduces thyroid hormone levels by lowering NIS and TSHr protein [37] and epidemiological studies have also shown that thyroid hormone deficiency affects cognitive development in children [38]. Additionally, a pregnant woman’s exposure to lead puts her developing fetus at risk of neurodevelopmental defects because lead can cross the placental barrier [39]. Moreover, children are more likely to ingest lead because of frequent hand to mouth contact [40], and they absorb 4–5 times as much ingested lead as adults from a given source [1]. Additionally, the blood-brain barrier is less developed in the developing brains of children; consequently, lead can more easily cross into several tissues, making children more sensitive to the toxic effects of lead exposure [41]. For CVD and DKD, PAFs were higher in adults over 60 years of age, which may be related to the cumulative effects of lead in the body. The widespread use of lead in the past may have led to higher lead exposure levels in older adults today [40]. Moreover, studies have shown that elevated lead levels in the body can increase the risk of hypertension and promote the development of atherosclerosis, thrombosis, and CVD [42], which may be associated with lead-induced high expression of renal angiotensin-converting enzyme [43]. Moreover, a study showed a significant positive association between blood lead levels and a risk of chronic kidney disease in the elderly, possibly due to oxidative stress in renal tubular and glomerular cells caused by lead [44]. Therefore, exposure to lead may lead to various disease risks in different populations, and this should be taken into account to effectively prevent and control lead exposure and reduce the disease burden.

Previously, males have been suggested to suffer from a higher disease burden resulting from lead exposure. However, no significant difference in disease burden between was found between males and females aged < 25 years in this study, possibly because males and females were equally exposed to lead in childhood and adolescence [45]. Additionally, the disease burden from lead exposure during this period was mainly due to MD, and there was no significant difference in the burden of MD between males and females. Moreover, between the age of 25–90 years, the burden was significantly higher in males than in females, and the diseases that contributed most to this difference were IHD and stroke. As adults, males are disproportionately employed in occupations associated with lead exposure, such as lead miners, construction workers, and mechanical workers [46]. Moreover, males are more likely to engage in activities such as smoking and alcohol consumption, which are risk factors for CVD and stroke, and smoking may also increase blood lead levels [47]. Therefore, males should reduce smoking and alcohol consumption, and exposure protection when working with lead should be improved to reduce the disease burden.

The burden of disease caused by lead exposure was negatively correlated with the SDI. We found that in areas with a high SDI, the burden was the lowest, and ASMRs and ASDRs declined the fastest. The opposite was observed in low and low-middle SDI areas, and some of them even showed an increase in ASMRs and ASDRs. This may be related to the different lead control strategies implemented globally, such as the fact that most high-income countries banned the use of leaded gasoline in the 1980s while almost all low- and middle-income countries continued to use leaded gasoline until as recently as 2002 [7]. Furthermore, low SDI areas failed to achieve strict environmental protection in waste lead-acid battery recycling, lead mining and smelting, and electronics recycling, resulting in much higher lead pollution than in areas with a high SDI [48]. Simultaneously, low SDI areas have relatively limited medical resources, lower socioeconomic status, and less disease awareness, while high SDI areas tend to have more advanced research methods and health care systems that can better diagnose and treat diseases; therefore, the disease burden in high SDI areas may be relatively low [2, 49]. Therefore, corresponding lead control strategies in low SDI countries should continue to be improved through measures such as strengthening blood lead monitoring in exposed populations and raising awareness of lead toxicity, while more multisectoral commitments are necessary to eliminate sources of lead and ultimately reduce the disease burden of lead exposure.

The spatial distribution of the burden resulting from the 13 diseases showed that, among the seven World Bank regions, the burden of all effects of lead exposure, as well as MD and DKD, was highest in South Asia. The high prevalence of and mortality resulting from these diseases in the region may be related; for example, the higher absolute burden of DKD in South Asia may be associated with a higher prevalence of insulin resistance and excess abdominal adipose tissue in the population of this region [50]. The highest absolute burden of CVD was observed in East Asia and the Pacific. In terms of the share of each disease, IHD accounted for the largest proportion in most regions. Stroke, another common CVD, had a greater burden in sub-Saharan Africa, East Asia, and the Pacific. As the largest country in East Asia, it has been reported that, in 2019, the incidence of strokes in China was close to 4 million, the number of resulting deaths exceeded 2 million, significantly higher than in the rest of the world [51], and that stroke was the primary cause of death in the country [52]. In sub-Saharan Africa and South Asia, the burden of MD caused by lead exposure was more severe than that of other diseases, whereas in Latin America and the Caribbean, DKD accounted for a higher proportion of the burden. Therefore, targeted lead control strategies and measures should be formulated according to the spatial distribution of the burden of various diseases caused by lead exposure.

In addition to directly causing diseases, lead exposure can indirectly threaten human health by affecting the survival of wildlife and contaminating water, soil, and air [53]. Therefore, the benefits of reducing lead exposure are substantial. The Pew Research Center estimated that the maximum potential benefit to be gained from preventing all lead exposure of children born in the United States in 2018 could be $84 billion [54]. The United Nations suggested that banning leaded gasoline could prevent more than 1.2 million premature deaths each year and boost children’s IQs, saving the global economy $2.45 trillion [55]. It has also been hypothesized that reducing lead exposure may lower the risk of premature death in individuals with diabetes [56]. Primary and secondary lead exposure prevention is essential to eliminate lead hazards in places where children live, learn, and play [57], and the same applies to the elderly. For adults with occupational lead exposure, companies should ensure engineering controls (ventilation and exhaust), administrative controls (limited hours of lead exposure), and personal protection through appropriate protective equipment [58]. Therefore, lead control is a difficult yet important task that requires continued improvements.

Finally, it is important to highlight the limitations of our study. Data were obtained from the GBD 2019 database, and the results were mainly estimated by a system dynamics model combined with a statistical model rather than real observations, inferring that the estimated results may be distorted. Furthermore, the GBD database included all data from 1990 to 2019, which may have influenced the results due to inconsistent diagnostic criteria of diseases across different periods. Moreover, comprehensive assessments of disease burdens should include economic, family, and social assessments, and future studies should therefore include multidimensional analyses to provide more accurate results. Additionally, it is important to translate research findings on the effects of lead exposure on human health into actions, such as developing public policies and guiding clinical decisions, aiming to reduce the burden of disease caused by lead exposure.

In future studies, the dose-response curves of lead exposure and specific types of diseases should be considered. Point-to-point quantitative results will aid more accurate assessments of the burden of lead exposure. Therefore, based on the spatio-temporal discrepancies of lead exposure sources and the specificity of susceptible populations, it would be more reasonable to compare the attributable burden of lead exposure among different populations from different regions. The results of the current study provide epidemiological evidence for targeted measures to control lead exposure and its attributable burden in countries worldwide.

## Conclusion

Although age-standardized mortality and DALY rates of lead exposure have decreased globally, deaths and DALY numbers have increased significantly in the context of population growth and aging. Continued reduction of lead exposure is an urgent priority for local and national governments, who should encourage stricter measures to combat lead pollution. Recommendations should be tailored to the increased susceptibility of infants, children, and the elderly, the specificity of IHD, stroke, and MD, and the particularity of low and middle SDI regions to reduce the global burden of lead exposure.

## Electronic supplementary material

Below is the link to the electronic supplementary material.

Supplementary Material 1

Supplementary Material 2

## Data Availability

All data generated or analyzed during this study are included in this published article and its supplementary information files. All data can be extracted from the online GBD repository, http://ghdx.healthdata.org/gbd-results-tool.
