# Supplementary material for: Patterns of global burden of 13 diseases attributable to lead exposure, 1990–2019
Source: BMC Public Health. 2023 Jun 12;23:1121. doi: 10.1186/s12889-023-15874-7 (PMC10262405; doi:10.1186/s12889-023-15874-7)
Supplement: Supplementary file 2 — Supplementary Material 2 [file 12889_2023_15874_MOESM2_ESM.docx]

| **Table S1** Number, rate and PAF of deaths in diabetes and kidney diseases attributable to lead exposure in 1990 and 2019, and the average annual percentage change in rate from 1990 to 2019, by age group. | | | | | | | | | |
| --- | --- | --- | --- | --- | --- | --- | --- | --- | --- |
| **Age group**  **（years）** | **1990** | | |  | **2019** | | |  | **1990-2019** |
|  | **Deaths**  **No.×10^3^**  **(95% UI)** | **Rate**  **per 100 000**  **No. (95% UI)** | **PAF, %**  **No.**  **(95% UI)** |  | **Deaths**  **No.×10^3^**  **(95% UI)** | **Rate**  **per 100 000**  **No. (95% UI)** | **PAF, %**  **No.**  **(95% UI)** |  | **AAPC in Rate**  **No. (95% CI)** |
| 25-29 | 0.20  (0.07, 0.37) | 0.04  (0.01, 0.08) | 1.10  (0.36, 2.07) |  | 0.08  (<0.01, 0.25) | 0.01  (<0.01, 0.04) | 0.38  (0.02, 1.13) |  | -3.94  (-4.36, -3.52) |
| 30-34 | 0.30  (0.13, 0.53) | 0.08  (0.03, 0.14) | 1.36  (0.59, 2.33) |  | 0.17  (0.02, 0.43) | 0.03  (<0.01, 0.07) | 0.56  (0.07, 1.35) |  | -3.41  (-3.74, -3.07) |
| 35-39 | 0.50  (0.25, 0.81) | 0.14  (0.07, 0.23) | 1.68  (0.84, 2.69) |  | 0.34  (0.08, 0.70) | 0.06  (0.02, 0.13) | 0.79  (0.20, 1.63) |  | -2.77  (-2.96, -2.58) |
| 40-44 | 0.72  (0.40, 1.08) | 0.25  (0.14, 0.38) | 1.85  (1.04, 2.79) |  | 0.65  (0.23, 1.18) | 0.13  (0.05, 0.24) | 0.98  (0.34, 1.78) |  | -2.16  (-2.36, -1.97) |
| 45-49 | 0.94  (0.55, 1.37) | 0.40  (0.24, 0.59) | 1.90  (1.15, 2.78) |  | 1.17  (0.53, 1.93) | 0.25  (0.11, 0.41) | 1.17  (0.53, 1.93) |  | -1.68  (-1.90, -1.46) |
| 50-54 | 1.44  (0.88, 2.05) | 0.68  (0.42, 0.96) | 1.90  (1.16, 2.70) |  | 2.24  (1.17, 3.44) | 0.51  (0.27, 0.79) | 1.39  (0.73, 2.13) |  | -0.91  (-1.28, -0.53) |
| 55-59 | 1.98  (1.27, 2.77) | 1.07  (0.68, 1.49) | 1.92  (1.22, 2.67) |  | 3.38  (1.92, 4.97) | 0.91  (0.52, 1.34) | 1.53  (0.89, 2.26) |  | -0.58  (-0.80, -0.36) |
| 60-64 | 2.56  (1.66, 3.55) | 1.59  (1.03, 2.21) | 1.85  (1.19, 2.55) |  | 4.75  (2.87, 6.83) | 1.52  (0.92, 2.19) | 1.62  (0.99, 2.32) |  | -0.14  (-0.59, 0.32) |
| 65-69 | 2.78  (1.79, 3.87) | 2.25  (1.45, 3.13) | 1.79  (1.16, 2.47) |  | 6.28  (3.96, 8.84) | 2.43  (1.53, 3.42) | 1.80  (1.15, 2.54) |  | 0.26  (-0.03, 0.56) |
| 70-74 | 2.85  (1.80, 4.05) | 3.37  (2.12, 4.79) | 1.81  (1.15, 2.54) |  | 7.22  (4.62, 10.11) | 3.86  (2.47, 5.40) | 1.96  (1.28, 2.70) |  | 0.46  (0.19, 0.72) |
| 75-79 | 2.82  (1.71, 4.10) | 4.59  (2.79, 6.68) | 1.69  (1.03, 2.43) |  | 7.81  (5.02, 10.86) | 6.15  (3.95, 8.54) | 2.04  (1.34, 2.83) |  | 1.01  (0.87, 1.15) |
| 80-84 | 2.16  (1.23, 3.25) | 6.14  (3.48, 9.22) | 1.62  (0.94, 2.41) |  | 7.63  (4.75, 10.85) | 9.04  (5.63, 12.85) | 2.05  (1.31, 2.87) |  | 1.35  (1.13, 1.57) |
| 85-89 | 1.38  (0.75, 2.11) | 9.17  (4.99, 14.00) | 1.72  (0.96, 2.58) |  | 6.24  (3.77, 9.02) | 14.36  (8.66, 20.74) | 2.17  (1.37, 3.08) |  | 1.57  (1.29, 1.85) |
| 90-94 | 0.56  (0.29, 0.89) | 12.81  (6.64, 20.12) | 1.70  (0.90, 2.60) |  | 3.43  (1.95, 5.14) | 20.37  (11.54, 30.48) | 2.10  (1.22, 3.09) |  | 1.62  (1.44, 1.8) |
| 95+ | 0.20  (0.10, 0.32) | 19.52  (9.94, 31.31) | 1.78  (0.94, 2.74) |  | 1.52  (0.79, 2.44) | 31.93  (16.62, 51.11) | 2.08  (1.14, 3.15) |  | 1.70  (1.59, 1.82) |

Note: No., number; Rate, deaths per 100,000 population; PAF, population attributable fraction; UI, uncertainty interval; CI, confidential interval; AAPC, average annual percentage change.

| **Table S2** Number, rate and PAF of DALYs in diabetes and kidney diseases attributable to lead exposure in 1990 and 2019, and the average annual percentage change in rate from 1990 to 2019, by age group. | | | | | | | | | |
| --- | --- | --- | --- | --- | --- | --- | --- | --- | --- |
| **Age group**  **（years）** | **1990** | | |  | **2019** | | |  | **1990-2019** |
|  | **DALYs**  **No.×10^4^**  **(95% UI)** | **Rate**  **per 100 000**  **No. (95% UI)** | **PAF, %**  **No.**  **(95% UI)** |  | **DALYs**  **No.×10^4^**  **(95% UI)** | **Rate**  **per 100 000**  **No. (95% UI)** | **PAF, %**  **No.**  **(95% UI)** |  | **AAPC in Rate**  **No. (95% CI)** |
| 25-29 | 1.57  (0.51, 3.06) | 3.54  (1.15, 6.91) | 0.95  (0.30, 1.85) |  | 0.70  (0.03, 2.12) | 1.15  (0.05, 3.51) | 0.29  (0.01, 0.89) |  | -3.79  (-4.03, -3.56) |
| 30-34 | 2.23  (0.94, 3.90) | 5.77  (2.43, 10.11) | 1.14  (0.47, 2.00) |  | 1.33  (0.17, 3.34) | 2.20  (0.28, 5.56) | 0.40  (0.05, 1.01) |  | -3.26  (-3.45, -3.08) |
| 35-39 | 3.27  (1.65, 5.28) | 9.27  (4.68, 14.97) | 1.34  (0.66, 2.18) |  | 2.28  (0.56, 4.91) | 4.22  (1.04, 9.08) | 0.54  (0.13, 1.15) |  | -2.65  (-2.86, -2.44) |
| 40-44 | 4.12  (2.27, 6.30) | 14.38  (7.93, 22.02) | 1.45  (0.79, 2.23) |  | 3.85  (1.34, 7.10) | 7.81  (2.72, 14.39) | 0.67  (0.23, 1.24) |  | -2.03  (-2.21, -1.84) |
| 45-49 | 4.76  (2.81, 7.08) | 20.47  (12.11, 30.48) | 1.45  (0.84, 2.16) |  | 6.16  (2.70, 10.52) | 12.99  (5.71, 22.20) | 0.79  (0.34, 1.36) |  | -1.56  (-1.75, -1.36) |
| 50-54 | 6.28  (3.85, 9.10) | 29.54  (18.12, 42.82) | 1.45  (0.87, 2.12) |  | 10.01  (5.20, 15.86) | 22.92  (11.89, 36.31) | 0.95  (0.48, 1.51) |  | -0.86  (-1.26, -0.46) |
| 55-59 | 7.54  (4.76, 10.62) | 40.65  (25.69, 57.27) | 1.47  (0.92, 2.10) |  | 13.07  (7.39, 19.55) | 35.23  (19.91, 52.69) | 1.06  (0.60, 1.62) |  | -0.51  (-0.74, -0.28) |
| 60-64 | 8.32  (5.32, 11.58) | 51.82  (33.09, 72.08) | 1.43  (0.91, 2.01) |  | 15.67  (9.31, 22.74) | 50.14  (29.78, 72.76) | 1.14  (0.67, 1.67) |  | -0.11  (-0.44, 0.22) |
| 65-69 | 7.61  (4.88, 10.59) | 61.60  (39.52, 85.76) | 1.40  (0.88, 1.98) |  | 17.40  (10.90, 24.62) | 67.27  (42.17, 95.20) | 1.28  (0.78, 1.83) |  | 0.34  (0.03, 0.65) |
| 70-74 | 6.42  (4.04, 9.16) | 76.01  (47.85, 108.34) | 1.44  (0.91, 2.07) |  | 16.54  (10.58, 23.21) | 88.41  (56.53, 124.07) | 1.43  (0.89, 2.03) |  | 0.52  (0.27, 0.77) |
| 75-79 | 5.06  (3.06, 7.34) | 82.53  (49.86, 119.68) | 1.38  (0.83, 2.03) |  | 14.30  (9.21, 19.99) | 112.52  (72.52, 157.37) | 1.57  (1.00, 2.22) |  | 1.11  (0.98, 1.24) |
| 80-84 | 3.04  (1.73, 4.61) | 86.31  (49.13, 130.99) | 1.35  (0.77, 2.02) |  | 10.83  (6.71, 15.49) | 128.25  (79.46, 183.54) | 1.65  (1.04, 2.34) |  | 1.43  (1.20, 1.65) |
| 85-89 | 1.48  (0.81, 2.29) | 98.23  (53.76, 151.94) | 1.44  (0.79, 2.19) |  | 6.68  (4.06, 9.71) | 153.68  (93.33, 223.23) | 1.80  (1.11, 2.59) |  | 1.57  (1.44, 1.70) |
| 90-94 | 0.47  (0.24, 0.74) | 105.75  (54.55, 167.46) | 1.47  (0.77, 2.28) |  | 2.79  (1.59, 4.15) | 165.40  (94.42, 246.38) | 1.79  (1.04, 2.65) |  | 1.56  (1.41, 1.71) |
| 95+ | 0.13  (0.07, 0.20) | 124.17  (63.65, 199.04) | 1.61  (0.84, 2.50) |  | 0.92  (0.49, 1.47) | 193.45  (103.38, 307.22) | 1.85  (1.01, 2.83) |  | 1.53  (1.43, 1.64) |
| Note: No., number; Rate, DALYs per 100,000 population; PAF, population attributable fraction; UI, uncertainty interval; CI, confidential interval; AAPC, average annual percentage change; DALYs, disability-adjusted life years. | | | | | | | | | |

| **Table S3** Number, rate and PAF of DALYs in mental disorders attributable to lead exposure in 1990 and 2019, and the average annual percentage change in rate from 1990 to 2019, by age group. | | | | | | | | | |
| --- | --- | --- | --- | --- | --- | --- | --- | --- | --- |
| **Age group**  **（years）** | **1990** | | |  | **2019** | | |  | **1990-2019** |
|  | **DALYs**  **No.×10^5^**  **(95% UI)** | **Rate**  **per 100 000**  **No. (95% UI)** | **PAF, %**  **No.**  **(95% UI)** |  | **DALYs**  **No.×10^5^**  **(95% UI)** | **Rate**  **per 100 000**  **No. (95% UI)** | **PAF, %**  **No.**  **(95% UI)** |  | **AAPC in Rate**  **No. (95% CI)** |
| <5 | 3.97  (1.75, 6.90) | 62.75  (27.76, 109.17) | 35.17  (18.38, 48.15) |  | 2.66  (1.09, 4.78) | 40.06  (16.41, 72.15) | 25.14  (12.42, 36.35) |  | -1.53  (-1.58, -1.48) |
| 5-9 | 3.89  (1.78, 6.78) | 66.50  (30.49, 115.79) | 14.31  (7.00, 21.94) |  | 3.05  (1.30, 5.44) | 46.63  (19.80, 83.04) | 10.29  (4.76, 16.16) |  | -1.22  (-1.27, -1.17) |
| 10-14 | 3.29  (1.49, 5.71) | 61.37  (27.84, 106.36) | 5.40  (2.54, 8.77) |  | 2.99  (1.31, 5.38) | 46.62  (20.38, 83.79) | 4.04  (1.79, 6.72) |  | -0.96  (-1.00, -0.91) |
| 15-19 | 2.90  (1.32, 5.06) | 55.86  (25.38, 97.34) | 3.53  (1.71, 5.76) |  | 2.86  (1.27, 5.15) | 46.14  (20.51, 83.12) | 2.89  (1.33, 4.85) |  | -0.67  (-0.69, -0.64) |
| 20-24 | 2.59  (1.18, 4.51) | 52.64  (23.92, 91.53) | 2.93  (1.43, 4.86) |  | 2.66  (1.18, 4.80) | 44.35  (19.64, 79.90) | 2.51  (1.17, 4.30) |  | -0.59  (-0.62, -0.56) |
| 25-29 | 2.10  (0.95, 3.63) | 47.35  (21.37, 81.95) | 2.39  (1.15, 4.10) |  | 2.38  (1.04, 4.25) | 39.27  (17.17, 70.15) | 2.03  (0.96, 3.54) |  | -0.64  (-0.73, -0.55) |
| 30-34 | 1.65  (0.73, 2.87) | 42.69  (18.94, 74.36) | 2.03  (0.97, 3.48) |  | 2.16  (0.94, 3.85) | 35.86  (15.60, 63.96) | 1.76  (0.83, 3.07) |  | -0.61  (-0.65, -0.57) |
| 35-39 | 1.36  (0.59, 2.37) | 38.45  (16.77, 67.31) | 1.77  (0.81, 3.04) |  | 1.88  (0.83, 3.34) | 34.73  (15.27, 61.66) | 1.61  (0.74, 2.77) |  | -0.36  (-0.4, -0.32) |
| 40-44 | 1.00  (0.44, 1.76) | 35.06  (15.41, 61.40) | 1.59  (0.72, 2.72) |  | 1.58  (0.70, 2.78) | 32.04  (14.20, 56.29) | 1.47  (0.68, 2.49) |  | -0.31  (-0.39, -0.23) |
| 45-49 | 0.76  (0.33, 1.34) | 32.72  (14.41, 57.67) | 1.49  (0.68, 2.54) |  | 1.36  (0.61, 2.38) | 28.79  (12.84, 50.18) | 1.34  (0.60, 2.25) |  | -0.46  (-0.55, -0.37) |
| 50-54 | 0.60  (0.26, 1.05) | 28.01  (12.20, 49.22) | 1.32  (0.59, 2.23) |  | 1.12  (0.50, 1.93) | 25.54  (11.41, 44.25) | 1.21  (0.55, 2.04) |  | -0.33  (-0.45, -0.21) |
| 55-59 | 0.46  (0.20, 0.80) | 24.57  (10.62, 42.97) | 1.19  (0.51, 2.01) |  | 0.84  (0.38, 1.45) | 22.65  (10.28, 39.09) | 1.08  (0.49, 1.86) |  | -0.30  (-0.39, -0.20) |
| 60-64 | 0.32  (0.14, 0.56) | 19.91  (8.48, 35.01) | 0.99  (0.43, 1.69) |  | 0.63  (0.28, 1.09) | 20.04  (9.01, 34.74) | 0.99  (0.45, 1.70) |  | 0.01  (-0.04, 0.07) |
| 65-69 | 0.18  (0.07, 0.32) | 14.56  (5.93, 25.98) | 0.76  (0.32, 1.32) |  | 0.45  (0.20, 0.78) | 17.36  (7.85, 30.17) | 0.91  (0.42, 1.55) |  | 0.60  (0.56, 0.65) |
| 70-74 | 0.06  (0.02, 0.12) | 7.60  (2.89, 13.99) | 0.43  (0.17, 0.76) |  | 0.27  (0.12, 0.47) | 14.64  (6.56, 25.17) | 0.82  (0.38, 1.37) |  | 2.28  (2.15, 2.41) |
| 75-79 | 0.03  (0.01, 0.06) | 4.90  (1.82, 9.14) | 0.29  (0.11, 0.52) |  | 0.15  (0.07, 0.26) | 12.07  (5.39, 20.54) | 0.72  (0.33, 1.23) |  | 3.14  (3.01, 3.28) |
| 80-84 | 0.02  (0.01, 0.03) | 4.26  (1.65, 7.91) | 0.27  (0.10, 0.49) |  | 0.08  (0.04, 0.14) | 9.66  (4.43, 16.27) | 0.62  (0.28, 1.07) |  | 2.87  (2.78, 2.95) |
| 85-89 | 0.01  (<0.01, 0.01) | 3.45  (1.33, 6.34) | 0.23  (0.09, 0.41) |  | 0.03  (0.01, 0.05) | 7.20  (3.18, 12.34) | 0.49  (0.22, 0.84) |  | 2.57  (2.49, 2.65) |
| 90-94 | <0.01  (<0.01, <0.01) | 2.76  (1.02, 5.10) | 0.19  (0.07, 0.35) |  | 0.01  (<0.01, 0.01) | 4.97  (2.09, 8.57) | 0.36  (0.16, 0.62) |  | 2.06  (1.95, 2.17) |
| 95+ | <0.01  (<0.01, <0.01) | 2.21  (0.84, 4.06) | 0.16  (0.06, 0.31) |  | <0.01  (<0.01, <0.01) | 2.84  (1.17, 5.08) | 0.22  (0.09, 0.40) |  | 0.92  (0.76, 1.07) |

Note: No., number; Rate, DALYs per 100,000 population; PAF, population attributable fraction; UI, uncertainty interval; CI, confidential interval; AAPC, average annual percentage change; DALYs, disability-adjusted life years.

| **Table S4** Number, rate and PAF of deaths in all causes attributable to lead exposure in 1990 and 2019, and the average annual percentage change in rate from 1990 to 2019, by age group. | | | | | | | | | |
| --- | --- | --- | --- | --- | --- | --- | --- | --- | --- |
| **Age group**  **（years）** | **1990** | | |  | **2019** | | |  | **1990-2019** |
|  | **Deaths**  **No.×10^3^**  **(95% UI)** | **Rate**  **per 100 000**  **No. (95% UI)** | **PAF, %**  **No.**  **(95% UI)** |  | **Number**  **No.×10^3^**  **(95% UI)** | **Rate**  **per 100 000**  **No. (95% UI)** | **PAF, %**  **No.**  **(95% UI)** |  | **AAPC in Rate**  **No. (95% CI)** |
| 25-29 | 3.09  (1.09, 5.84) | 0.70  (0.25, 1.32) | 0.37  (0.13, 0.70) |  | 1.09  (0.06, 3.17) | 0.18  (0.01, 0.52) | 0.14  (0.01, 0.40) |  | -4.54  (-5.05, -4.02) |
| 30-34 | 5.29  (2.36, 9.07) | 1.37  (0.61, 2.35) | 0.60  (0.26, 1.02) |  | 2.66  (0.39, 6.39) | 0.44  (0.07, 1.06) | 0.27  (0.04, 0.65) |  | -3.81  (-4.16, -3.46) |
| 35-39 | 9.32  (4.83, 14.57) | 2.64  (1.37, 4.13) | 0.89  (0.46, 1.41) |  | 5.45  (1.49, 10.72) | 1.01  (0.28, 1.98) | 0.47  (0.13, 0.92) |  | -3.23  (-3.58, -2.88) |
| 40-44 | 14.95  (8.43, 22.47) | 5.22  (2.94, 7.85) | 1.29  (0.73, 1.93) |  | 11.07  (4.11, 19.53) | 2.24  (0.83, 3.96) | 0.77  (0.28, 1.35) |  | -2.82  (-2.94, -2.71) |
| 45-49 | 22.82  (13.68, 32.98) | 9.82  (5.88, 14.19) | 1.70  (1.02, 2.46) |  | 22.01  (10.34, 35.20) | 4.64  (2.18, 7.43) | 1.18  (0.55, 1.87) |  | -2.56  (-2.71, -2.42) |
| 50-54 | 37.65  (23.42, 52.93) | 17.71  (11.02, 24.90) | 2.00  (1.24, 2.80) |  | 41.59  (22.92, 62.17) | 9.52  (5.25, 14.23) | 1.63  (0.89, 2.40) |  | -2.08  (-2.42, -1.74) |
| 55-59 | 52.46  (33.64, 73.11) | 28.29  (18.15, 39.43) | 2.17  (1.40, 3.01) |  | 60.50  (35.74, 86.54) | 16.31  (9.63, 23.32) | 1.87  (1.11, 2.63) |  | -1.92  (-2.16, -1.68) |
| 60-64 | 68.09  (43.45, 95.59) | 42.39  (27.05, 59.50) | 2.14  (1.38, 3.00) |  | 83.69  (52.02, 117.43) | 26.78  (16.65, 37.57) | 2.03  (1.25, 2.82) |  | -1.55  (-1.84, -1.26) |
| 65-69 | 71.69  (44.37, 102.27) | 58.05  (35.93, 82.82) | 2.00  (1.24, 2.87) |  | 106.51  (67.28, 149.05) | 41.19  (26.02, 57.64) | 2.11  (1.34, 2.93) |  | -1.18  (-1.36, -0.99) |
| 70-74 | 71.60  (42.81, 104.88) | 84.72  (50.65, 124.10) | 1.86  (1.12, 2.73) |  | 122.24  (78.62, 171.92) | 65.34  (42.02, 91.89) | 2.13  (1.37, 2.96) |  | -0.88  (-1.00, -0.77) |
| 75-79 | 70.85  (39.23, 107.22) | 115.56  (63.99, 174.89) | 1.68  (0.94, 2.55) |  | 133.30  (85.40, 186.13) | 104.92  (67.21, 146.50) | 2.19  (1.42, 3.06) |  | -0.32  (-0.46, -0.18) |
| 80-84 | 52.07  (25.69, 83.59) | 147.83  (72.93, 237.35) | 1.39  (0.69, 2.24) |  | 132.36  (79.32, 196.34) | 156.78  (93.95, 232.57) | 1.96  (1.17, 2.90) |  | 0.27  (0.04, 0.49) |
| 85-89 | 32.14  (15.20, 52.94) | 213.28  (100.85, 351.29) | 1.34  (0.64, 2.20) |  | 105.58  (61.18, 159.63) | 242.82  (140.70, 367.13) | 1.95  (1.13, 2.91) |  | 0.45  (0.26, 0.64) |
| 90-94 | 13.13  (5.86, 22.20) | 297.91  (133.03, 503.89) | 1.33  (0.59, 2.25) |  | 52.84  (28.17, 82.83) | 313.45  (167.13, 491.33) | 1.71  (0.91, 2.68) |  | 0.21  (0.02, 0.40) |
| 95+ | 4.70  (2.03, 7.97) | 456.10  (197.23, 773.79) | 1.40  (0.60, 2.37) |  | 20.82  (10.21, 34.34) | 436.26  (213.94, 719.42) | 1.53  (0.75, 2.51) |  | -0.13  (-0.22, -0.04) |
| Note: No., number; Rate, deaths per 100,000 population; PAF, population attributable fraction; UI, uncertainty interval; CI, confidential interval; AAPC, average annual percentage change. | | | | | | | | | |

| **Table S5** Number, rate and PAF of DALYs in all causes attributable to lead exposure in 1990 and 2019, and the average annual percentage change in rate from 1990 to 2019, by age group. | | | | | | | | | |
| --- | --- | --- | --- | --- | --- | --- | --- | --- | --- |
| **Age group**  **（years）** | **1990** | | |  | **2019** | | |  | **1990-2019** |
|  | **Number**  **No.×10^5^**  **(95% UI)** | **Rate**  **per 100 000**  **No. (95% UI)** | **PAF, %**  **No.**  **(95% UI)** |  | **Number**  **No.×10^5^**  **(95% UI)** | **Rate**  **per 100 000**  **No. (95% UI)** | **PAF, %**  **No.**  **(95% UI)** |  | **AAPC in Rate**  **No. (95% CI)** |
| <5 | 3.97  (1.75, 6.90) | 62.75  (27.76, 109.17) | 0.04  (0.02, 0.06) |  | 2.66  (1.09, 4.78) | 40.06  (16.41, 72.15) | 0.06  (0.02, 0.10) |  | -1.53  (-1.58, -1.48) |
| 5-9 | 3.89  (1.78, 6.78) | 66.50  (30.49, 115.79) | 0.41  (0.19, 0.68) |  | 3.05  (1.30, 5.44) | 46.63  (19.80, 83.04) | 0.51  (0.23, 0.83) |  | -1.22  (-1.27, -1.17) |
| 10-14 | 3.29  (1.49, 5.71) | 61.37  (27.84, 106.36) | 0.51  (0.24, 0.83) |  | 2.99  (1.31, 5.38) | 46.62  (20.38, 83.79) | 0.52  (0.23, 0.85) |  | -0.96  (-1.00, -0.91) |
| 15-19 | 2.90  (1.32, 5.06) | 55.86  (25.38, 97.34) | 0.34  (0.16, 0.56) |  | 2.86  (1.27, 5.15) | 46.14  (20.51, 83.12) | 0.37  (0.17, 0.62) |  | -0.67  (-0.69, -0.64) |
| 20-24 | 2.59  (1.18, 4.51) | 52.64  (23.92, 91.53) | 0.27  (0.12, 0.44) |  | 2.66  (1.18, 4.80) | 44.35  (19.64, 79.90) | 0.28  (0.13, 0.47) |  | -0.59  (-0.62, -0.56) |
| 25-29 | 4.20  (2.20, 6.59) | 94.93  (49.78, 148.82) | 0.44  (0.24, 0.68) |  | 3.14  (1.41, 5.27) | 51.89  (23.25, 87.08) | 0.30  (0.14, 0.50) |  | -2.07  (-2.21, -1.92) |
| 30-34 | 4.90  (2.74, 7.55) | 126.96  (71.02, 195.66) | 0.53  (0.30, 0.81) |  | 3.82  (1.67, 6.58) | 63.46  (27.70, 109.42) | 0.33  (0.15, 0.55) |  | -2.36  (-2.51, -2.21) |
| 35-39 | 6.51  (3.80, 9.68) | 184.68  (107.76, 274.33) | 0.68  (0.40, 1.01) |  | 4.93  (2.25, 8.30) | 91.09  (41.60, 153.51) | 0.40  (0.19, 0.66) |  | -2.38  (-2.60, -2.15) |
| 40-44 | 8.44  (5.05, 12.17) | 294.65  (176.45, 425.19) | 0.92  (0.55, 1.35) |  | 7.17  (3.42, 11.71) | 145.30  (69.38, 237.24) | 0.56  (0.27, 0.91) |  | -2.39  (-2.48, -2.30) |
| 45-49 | 10.86  (6.74, 15.36) | 467.12  (290.16, 661.08) | 1.22  (0.76, 1.75) |  | 11.27  (5.84, 17.39) | 237.84  (123.36, 367.10) | 0.79  (0.41, 1.24) |  | -2.34  (-2.51, -2.16) |
| 50-54 | 15.29  (9.73, 21.31) | 719.18  (457.63, 1002.35) | 1.48  (0.93, 2.09) |  | 17.65  (10.09, 25.92) | 404.00  (230.91, 593.46) | 1.10  (0.62, 1.63) |  | -1.93  (-2.25, -1.61) |
| 55-59 | 18.38  (11.94, 25.31) | 991.42  (643.76, 1365.05) | 1.67  (1.08, 2.32) |  | 21.90  (13.33, 31.09) | 590.36  (359.35, 838.02) | 1.31  (0.79, 1.88) |  | -1.81  (-2.03, -1.58) |
| 60-64 | 20.32  (13.01, 28.17) | 1264.99  (809.84, 1753.45) | 1.70  (1.10, 2.39) |  | 25.73  (16.10, 35.75) | 823.42  (515.12, 1143.91) | 1.48  (0.93, 2.08) |  | -1.45  (-1.73, -1.16) |
| 65-69 | 17.97  (11.18, 25.71) | 1454.98  (905.17, 2082.19) | 1.62  (1.01, 2.32) |  | 27.50  (17.55, 38.43) | 1063.54  (678.51, 1486.08) | 1.58  (1.01, 2.22) |  | -1.07  (-1.25, -0.90) |
| 70-74 | 14.62  (8.80, 21.50) | 1730.28  (1041.76, 2544.26) | 1.55  (0.93, 2.28) |  | 25.72  (16.62, 36.03) | 1374.53  (888.35, 1926.01) | 1.66  (1.05, 2.34) |  | -0.78  (-0.89, -0.66) |
| 75-79 | 11.43  (6.43, 17.32) | 1864.74  (1048.36, 2824.90) | 1.41  (0.79, 2.14) |  | 22.05  (14.17, 30.92) | 1735.55  (1114.97, 2434.00) | 1.74  (1.13, 2.45) |  | -0.24  (-0.37, -0.11) |
| 80-84 | 6.44  (3.20, 10.26) | 1827.67  (909.44, 2914.50) | 1.18  (0.59, 1.92) |  | 16.60  (9.86, 24.36) | 1966.49  (1168.25, 2885.25) | 1.60  (0.96, 2.38) |  | 0.32  (0.09, 0.55) |
| 85-89 | 3.03  (1.44, 4.96) | 2009.06  (955.53, 3289.26) | 1.15  (0.55, 1.90) |  | 10.04  (5.81, 14.98) | 2308.36  (1336.99, 3445.23) | 1.61  (0.94, 2.42) |  | 0.48  (0.30, 0.66) |
| 90-94 | 0.96  (0.43, 1.60) | 2170.94  (970.79, 3632.00) | 1.14  (0.51, 1.94) |  | 3.88  (2.08, 6.01) | 2299.58  (1233.08, 3567.66) | 1.42  (0.76, 2.23) |  | 0.21  (-0.02, 0.43) |
| 95+ | 0.26  (0.11, 0.44) | 2520.79  (1089.41, 4264.72) | 1.19  (0.52, 2.05) |  | 1.15  (0.57, 1.87) | 2402.85  (1189.58, 3921.27) | 1.29  (0.63, 2.11) |  | -0.15  (-0.24, -0.05) |

Note: No., number; Rate, DALYs per 100,000 population; PAF, population attributable fraction; UI, uncertainty interval; CI, confidential interval; AAPC, average annual percentage change; DALYs, disability-adjusted life years.

| **Table S6** Deaths and ASMR in all causes attributable to lead exposure in 1990 and 2019, and its average annual percentage change from 1990 to 2019, by location. | | | | | | | |
| --- | --- | --- | --- | --- | --- | --- | --- |
| **Characteristics** | **1990** | |  | **2019** | |  | **1990-2019** |
|  | **Deaths**  **No.×10^5^ (95% UI)** | **ASMR per 100000**  **No. (95% UI)** |  | **Deaths**  **No.×10^5^ (95% UI)** | **ASMR per 100000**  **No. (95% UI)** |  | **AAPC in ASMR**  **No. (95% CI)** |
| **SDI region** |  |  |  |  |  |  |  |
| High SDI | 0.60  (0.19, 1.07) | 5.75  (1.86, 10.31) |  | 0.52  (0.14, 1.00) | 2.31  (0.60, 4.51) |  | -3.10  (-3.28, -2.91) |
| High-middle SDI | 1.06  (0.51, 1.73) | 11.04  (5.22, 18.21) |  | 1.56  (0.78, 2.48) | 7.92  (3.94, 12.58) |  | -1.14  (-1.39, -0.89) |
| Middle SDI | 1.76  (1.10, 2.49) | 20.13  (12.38, 28.86) |  | 3.32  (2.04, 4.70) | 15.75  (9.69, 22.38) |  | -0.86  (-1.01, -0.71) |
| Low-middle SDI | 1.36  (0.96, 1.81) | 25.55  (17.63, 34.74) |  | 2.64  (1.81, 3.53) | 22.24  (15.26, 29.65) |  | -0.42  (-0.53, -0.32) |
| Low SDI | 0.52  (0.35, 0.72) | 25.00  (16.97, 34.86) |  | 0.97  (0.66, 1.30) | 22.73  (15.63, 30.72) |  | -0.36  (-0.45, -0.27) |
| **GBD region** |  |  |  |  |  |  |  |
| High-income Asia Pacific | 0.06  (0.01, 0.12) | 3.52  (0.59, 7.02) |  | 0.06  (0.01, 0.13) | 0.98  (0.13, 2.20) |  | -4.34  (-4.48, -4.20) |
| High-income North America | 0.26  (0.11, 0.44) | 7.20  (2.88, 11.98) |  | 0.22  (0.06, 0.40) | 3.01  (0.81, 5.71) |  | -2.98  (-3.07, -2.89) |
| Western Europe | 0.33  (0.11, 0.59) | 5.65  (1.79, 10.13) |  | 0.29  (0.10, 0.54) | 2.51  (0.80, 4.67) |  | -2.77  (-2.98, -2.56) |
| Australasia | 0.02  (0.01, 0.03) | 8.97  (4.24, 14.11) |  | 0.02  (0.01, 0.03) | 3.39  (1.58, 5.36) |  | -3.26  (-3.53, -2.99) |
| Southern Latin America | 0.02  (<0.01, 0.04) | 4.15  (0.17, 9.34) |  | 0.02  (<0.01, 0.05) | 2.57  (0.35, 5.51) |  | -1.63  (-1.81, -1.45) |
| Andean Latin America | 0.01  (0.01, 0.02) | 8.02  (3.75, 12.47) |  | 0.03  (0.02, 0.05) | 5.56  (2.83, 8.72) |  | -1.23  (-1.64, -0.81) |
| Tropical Latin America | 0.11  (0.05, 0.16) | 13.23  (6.54, 20.30) |  | 0.14  (0.07, 0.23) | 6.17  (2.99, 9.75) |  | -2.56  (-2.67, -2.45) |
| Central Latin America | 0.11  (0.07, 0.15) | 14.91  (9.82, 20.48) |  | 0.25  (0.16, 0.36) | 11.32  (7.21, 16.24) |  | -0.97  (-1.22, -0.72) |
| Caribbean | 0.04  (0.03, 0.06) | 17.68  (10.99, 24.92) |  | 0.07  (0.05, 0.10) | 14.05  (8.76, 19.99) |  | -0.73  (-1.11, -0.36) |
| Eastern Europe | 0.09  (<0.01, 0.27) | 3.65  (0.01, 10.94) |  | 0.12  (<0.01, 0.34) | 3.54  (0.08, 9.78) |  | -0.09  (-0.71, 0.54) |
| Central Europe | 0.14  (0.03, 0.26) | 10.30  (2.47, 19.13) |  | 0.15  (0.05, 0.27) | 6.71  (2.03, 12.19) |  | -1.49  (-1.75, -1.23) |
| Central Asia | 0.05  (0.01, 0.09) | 12.28  (3.71, 21.48) |  | 0.08  (0.03, 0.15) | 15.41  (5.79, 26.14) |  | 0.83  (0.48, 1.18) |
| North Africa and Middle East | 0.49  (0.32, 0.66) | 32.34  (21.20, 44.72) |  | 0.84  (0.53, 1.18) | 23.36  (15.11, 33.28) |  | -1.10  (-1.32, -0.87) |
| South Asia | 1.41  (1.02, 1.86) | 28.66  (20.42, 37.95) |  | 2.88  (2.01, 3.83) | 23.75  (16.68, 31.48) |  | -0.62  (-0.80, -0.45) |
| Southeast Asia | 0.25  (0.11, 0.41) | 10.88  (4.74, 17.79) |  | 0.51  (0.23, 0.83) | 9.65  (4.50, 15.60) |  | -0.41  (-0.47, -0.35) |
| East Asia | 1.64  (1.05, 2.30) | 22.64  (14.29, 32.29) |  | 2.87  (1.79, 4.08) | 16.30  (10.03, 23.20) |  | -1.14  (-1.29, -0.99) |
| Oceania | <0.01  (<0.01, <0.01) | 5.24  (0.61, 12.56) |  | <0.01  (<0.01, 0.01) | 5.07  (0.65, 12.12) |  | -0.11  (-0.20, -0.02) |
| Western Sub-Saharan Africa | 0.09  (0.04, 0.14) | 11.33  (5.19, 18.11) |  | 0.17  (0.09, 0.26) | 11.20  (5.98, 17.08) |  | -0.03  (-0.08, 0.01) |
| Eastern Sub-Saharan Africa | 0.13  (0.08, 0.19) | 20.32  (12.47, 29.79) |  | 0.18  (0.10, 0.27) | 14.97  (8.76, 22.05) |  | -1.06  (-1.16, -0.97) |
| Central Sub-Saharan Africa | 0.03  (0.01, 0.05) | 14.99  (7.09, 24.68) |  | 0.06  (0.03, 0.10) | 14.66  (7.61, 23.85) |  | -0.07  (-0.16, 0.02) |
| Southern Sub-Saharan Africa | 0.02  (0.01, 0.03) | 7.79  (3.26, 12.98) |  | 0.04  (0.02, 0.06) | 8.21  (3.56, 13.80) |  | 0.17  (-0.19, 0.53) |

Note: No., number; ASMR, age-standardized mortality rate; UI, uncertainty interval; AAPC, average annual percentage change; CI, confidential interval.

| **Table S7** DALYs and ASDR in all causes attributable to lead exposure in 1990 and 2019, and its average annual percentage change from 1990 to 2019, by location. | | | | | | | |
| --- | --- | --- | --- | --- | --- | --- | --- |
| **Characteristics** | **1990** | |  | **2019** | |  | **1990-2019** |
|  | **DALYs**  **No.×10^5^ (95% UI)** | **ASDR per 100 000**  **No. (95% UI)** |  | **DALYs**  **No.×10^5^ (95% UI)** | **ASDR per 100 000**  **No. (95% UI)** |  | **AAPC in ASDR**  **No. (95% CI)** |
| **SDI region** |  |  |  |  |  |  |  |
| High SDI | 12.37  (4.48, 21.60) | 122.76  (45.61, 213.29) |  | 8.58  (2.57, 16.43) | 46.49  (14.45, 88.60) |  | -3.29  (-3.37, -3.21) |
| High-middle SDI | 27.61  (14.54, 42.85) | 258.26  (135.59, 399.91) |  | 30.55  (15.73, 47.97) | 155.13  (81.03, 242.98) |  | -1.74  (-1.93, -1.54) |
| Middle SDI | 53.64  (35.13, 73.14) | 480.45  (313.65, 656.74) |  | 75.81  (46.85, 106.84) | 317.54  (199.19, 445.11) |  | -1.44  (-1.54, -1.34) |
| Low-middle SDI | 48.35  (35.07, 62.66) | 680.60  (493.13, 877.72) |  | 71.43  (50.12, 93.99) | 510.03  (360.25, 669.85) |  | -0.95  (-1.08, -0.83) |
| Low SDI | 18.20  (12.77, 24.27) | 639.49  (449.87, 855.57) |  | 30.28  (20.87, 40.05) | 517.68  (365.68, 680.93) |  | -0.74  (-0.84, -0.64) |
| **GBD region** |  |  |  |  |  |  |  |
| High-income Asia Pacific | 1.48  (0.39, 2.86) | 76.47  (20.48, 146.46) |  | 0.89  (0.16, 1.98) | 19.07  (3.81, 43.80) |  | -4.69  (-4.85, -4.53) |
| High-income North America | 5.33  (2.22, 8.69) | 156.17  (65.08, 255.11) |  | 3.64  (1.03, 6.97) | 58.81  (17.12, 112.92) |  | -3.31  (-3.38, -3.25) |
| Western Europe | 6.49  (2.47, 11.18) | 118.60  (46.47, 203.16) |  | 4.15  (1.56, 7.47) | 45.41  (17.76, 81.00) |  | -3.27  (-3.4, -3.15) |
| Australasia | 0.41  (0.20, 0.62) | 177.00  (86.69, 268.62) |  | 0.30  (0.14, 0.48) | 59.89  (28.88, 96.52) |  | -3.65  (-3.87, -3.43) |
| Southern Latin America | 0.53  (0.12, 1.03) | 112.68  (25.01, 223.98) |  | 0.48  (0.13, 0.94) | 59.78  (16.64, 115.79) |  | -2.17  (-2.29, -2.05) |
| Andean Latin America | 0.44  (0.23, 0.67) | 190.06  (97.38, 289.36) |  | 0.64  (0.32, 1.02) | 113.21  (57.88, 180.26) |  | -1.77  (-2.1, -1.44) |
| Tropical Latin America | 2.96  (1.45, 4.55) | 304.02  (148.67, 461.44) |  | 3.01  (1.39, 4.83) | 126.24  (59.47, 200.35) |  | -2.98  (-3.08, -2.87) |
| Central Latin America | 2.73  (1.81, 3.69) | 312.93  (206.66, 420.67) |  | 4.98  (3.06, 7.12) | 213.87  (132.91, 306.27) |  | -1.33  (-1.45, -1.21) |
| Caribbean | 1.09  (0.69, 1.50) | 408.38  (256.53, 562.53) |  | 1.55  (0.94, 2.24) | 301.24  (183.33, 434.41) |  | -1.00  (-1.31, -0.68) |
| Eastern Europe | 2.27  (0.19, 6.22) | 86.06  (8.55, 231.64) |  | 2.52  (0.21, 6.70) | 77.97  (8.97, 203.81) |  | -0.25  (-1.08, 0.59) |
| Central Europe | 3.18  (0.90, 5.69) | 223.12  (64.86, 399.18) |  | 2.56  (0.80, 4.63) | 122.19  (39.87, 221.24) |  | -2.07  (-2.28, -1.85) |
| Central Asia | 1.29  (0.46, 2.23) | 267.39  (91.17, 468.97) |  | 1.94  (0.67, 3.39) | 284.95  (104.73, 493.70) |  | 0.26  (0.10, 0.42) |
| North Africa and Middle East | 14.99  (10.52, 19.91) | 773.59  (530.72, 1029.37) |  | 21.43  (13.95, 29.56) | 489.27  (320.52, 669.63) |  | -1.55  (-1.63, -1.47) |
| South Asia | 53.74  (39.51, 68.55) | 774.47  (579.28, 978.54) |  | 84.39  (60.25, 110.57) | 576.94  (414.10, 751.70) |  | -1.00  (-1.37, -0.63) |
| Southeast Asia | 9.53  (5.04, 14.28) | 307.23  (158.58, 466.63) |  | 13.45  (6.25, 22.24) | 220.16  (106.08, 357.47) |  | -1.14  (-1.18, -1.1) |
| East Asia | 45.27  (29.74, 62.10) | 506.13  (327.33, 694.37) |  | 57.77  (35.95, 81.22) | 294.27  (183.88, 415.64) |  | -1.85  (-1.99, -1.72) |
| Oceania | 0.06  (0.02, 0.12) | 143.15  (33.54, 320.23) |  | 0.10  (0.03, 0.24) | 127.28  (29.01, 294.54) |  | -0.42  (-0.46, -0.38) |
| Western Sub-Saharan Africa | 2.75  (1.44, 4.19) | 271.57  (135.51, 423.27) |  | 4.90  (2.66, 7.38) | 236.76  (130.45, 360.84) |  | -0.46  (-0.53, -0.39) |
| Eastern Sub-Saharan Africa | 4.18  (2.65, 5.91) | 478.34  (302.73, 681.87) |  | 5.25  (3.02, 7.69) | 300.86  (178.49, 437.82) |  | -1.60  (-1.7, -1.51) |
| Central Sub-Saharan Africa | 0.94  (0.49, 1.45) | 358.38  (179.53, 562.32) |  | 1.84  (0.95, 2.87) | 314.37  (166.43, 493.17) |  | -0.44  (-0.56, -0.33) |
| Southern Sub-Saharan Africa | 0.59  (0.28, 0.96) | 192.71  (88.49, 314.30) |  | 0.97  (0.44, 1.59) | 171.52  (77.35, 282.06) |  | -0.42  (-0.78, -0.05) |

Note: No., number; DALYs, disability-adjusted life years; ASDR, age-standardized disability-adjusted life years rate; UI, uncertainty interval; AAPC, average annual percentage change; CI, confidential interval.

**Table S8** Top 10 countries or territories with the highest deaths, ASMR, AAPC of ASMR and the lowest AAPC of ASMR attributable to lead exposure in 2019.

| **Location** | **Deaths**  **(95% UI)** | **Location** | **ASMR**  **(95% UI)** | **Location** | **The highest AAPC of ASMR**  **(95% CI)** | **Location** | **The lowest AAPC of ASMR**  **(95% CI)** |
| --- | --- | --- | --- | --- | --- | --- | --- |
| China | 282200.38  (177008.69, 400747.70) | Bulgaria | 26.18  (6.55, 51.31) | Uzbekistan | 2.58  (2.27, 2.90) | Republic of Korea | -5.20  (-5.48, -4.93) |
| India | 232512.92  (161336.02, 310527.39) | Bosnia and Herzegovina | 22.97  (12.47, 35.71) | Tajikistan | 2.00  (1.61, 2.39) | Japan | -4.14  (-4.41, -3.86) |
| Bangladesh | 30776.58  (20283.85, 42149.41) | Malta | 22.43  (15.10, 31.63) | Philippines | 1.89  (1.70, 2.08) | United Kingdom | -3.85  (-4.11, -3.59) |
| Indonesia | 27399.71  (13714.15, 43047.23) | Afghanistan | 22.35  (15.72, 30.07) | Azerbaijan | 1.48  (1.08, 1.88) | Singapore | -3.84  (-3.98, -3.69) |
| Pakistan | 21167.44  (12736.37, 31148.02) | Yemen | 21.62  (15.19, 29.62) | Timor-Leste | 1.18  (1.07, 1.29) | Ireland | -3.82  (-3.99, -3.64) |
| United States of America | 20799.96  (6129.03, 38252.27) | Saint Vincent and the Grenadines | 20.92  (13.10, 30.01) | Lesotho | 1.16  (1.07, 1.25) | Israel | -3.81  (-4.18, -3.44) |
| Egypt | 19161.02  (11627.19, 28808.24) | China | 19.84  (12.44, 28.18) | Kyrgyzstan | 1.11  (0.29, 1.93) | Luxembourg | -3.80  (-3.96, -3.64) |
| Iran (Islamic Republic of) | 14878.36  (10365.97, 20243.17) | Romania | 19.62  (6.73, 34.86) | Burkina Faso | 1.07  (0.92, 1.22) | Norway | -3.67  (-3.98, -3.36) |
| Brazil | 13743.53  (6507.54, 21880.25) | Egypt | 19.34  (11.74, 29.08) | Honduras | 0.99  (0.60, 1.38) | Portugal | -3.65  (-3.93, -3.36) |
| Mexico | 12211.58  (7624.42, 17578.67) | Bangladesh | 19.32  (12.74, 26.47) | Chad | 0.96  (0.84, 1.08) | Iceland | -3.43  (-3.68, -3.18) |

Note: ASMR, age-standardized mortality rate; UI, uncertainty interval; CI, confidential interval; AAPC, average annual percentage change.

**Table S9** Top 10 countries or territories with the highest DALYs, ASDR, AAPC of ASDR and the lowest AAPC of ASDR attributable to lead exposure in 2019.

| **Location** | **DALYs**  **(95% UI)** | **Location** | **ASDR**  **(95% UI)** | **Location** | **The highest AAPC of ASDR**  **(95% CI)** | **Location** | **The lowest AAPC of ASDR**  **(95% CI)** |
| --- | --- | --- | --- | --- | --- | --- | --- |
| India | 6964017.28  (4950956.15, 9096939.64) | Afghanistan | 741.82  (529.20, 994.50) | Uzbekistan | 1.52  (1.25, 1.78) | Republic of Korea | -6.10  (-6.28, -5.92) |
| China | 5657903.94  (3537378.79, 7948508.71) | Yemen | 628.97  (441.86, 837.82) | Philippines | 1.23  (1.02, 1.44) | Singapore | -4.56  (-4.70, -4.42) |
| Indonesia | 711529.58  (349816.40, 1156655.15) | Egypt | 509.71  (316.08, 757.51) | Tajikistan | 1.09  (0.71, 1.46) | Ireland | -4.54  (-4.68, -4.40) |
| Bangladesh | 690420.96  (460612.79, 943665.27) | Sudan | 508.14  (353.33, 702.16) | Lesotho | 0.69  (0.59, 0.79) | Luxembourg | -4.28  (-4.45, -4.12) |
| Pakistan | 678025.93  (411602.88, 982016.61) | India | 500.75  (356.00, 654.12) | Chad | 0.58  (0.46, 0.69) | United Kingdom | -4.24  (-4.37, -4.11) |
| Egypt | 504963.78  (313142.34, 750465.12) | Haiti | 481.92  (301.34, 703.29) | Democratic People's Republic of Korea | 0.58  (0.53, 0.62) | Japan | -4.12  (-4.15, -4.10) |
| United States of America | 347834.96  (99195.88, 659421.54) | Syrian Arab Republic | 469.05  (279.42, 689.62) | Burkina Faso | 0.54  (0.45, 0.63) | Norway | -4.10  (-4.36, -3.84) |
| Iran (Islamic Republic of) | 320434.86  (227955.70, 419538.75) | Bulgaria | 436.89  (102.82, 872.95) | Guinea | 0.52  (0.41, 0.64) | Israel | -4.00  (-4.29, -3.70) |
| Brazil | 291910.71  (134307.27, 468526.23) | Bangladesh | 433.52  (289.22, 592.53) | Pakistan | 0.36  (0.30, 0.41) | Portugal | -3.99  (-4.23, -3.74) |
| Afghanistan | 283951.10  (202566.36, 380669.75) | Bosnia and Herzegovina | 414.75  (221.34, 645.86) | Zimbabwe | 0.34  (0.13, 0.56) | Denmark | -3.98  (-4.15, -3.82) |

Note: DALYs, disability-adjusted life years; ASDR, age-standardized disability-adjusted life years rate; UI, uncertainty interval; CI, confidential interval; AAPC, average annual percentage change.

**Fig. S1** PAF of specific GBD level-three diseases in ASDR attributable to lead exposure by age group and by region for both sexes in 2019. (A) By age group. (B) By region. ASDR, age-standardized disability-adjusted life year rate; GBD, Global Burden of Disease Study; SDI, socio-demographic index.

**Fig. S2** Age-specific numbers (bar plot) and rates (line plot) of DALYs attributable to lead exposure in 2019 by sex. DALYs, disability-adjusted life years.

**Fig. S3** Global DALYs burden attributable to lead exposure for both sexes. (A) Number of DALYs in 2019, and pie plots depict the proportion of specific GBD level-three diseases by World Bank Region (the number represents the number of specific cause). (B) ASDR in 2019. (C) AAPC of ASDR from 1990 to 2019. DALYs, disability-adjusted life years; ASDR, age-standardized DALYs rate; AAPC, average annual percentage change; GBD, Global Burden of Disease Study.

**Fig. S4** Numbers of deaths and DALYs of specific GBD level-three diseases attributable to lead exposure by age group for both sexes in 2019. (A) Deaths. (B) DALYs. DALYs, disability-adjusted life years; GBD, Global Burden of Disease Study.

**Fig. S5** Number of all-age DALYs and ASDR attributable lead exposure by sex, 1990–2019. (A) DALYs. (B) ASDR. ASDR, age-standardized disability-adjusted life year rate.

**Fig. S6** The factors associated with the AAPC of ASDR attributable to lead exposure from 1990 to 2019, both sexes, at the national level. (A) The corresponding ASDR in 2019; (B) Socio-demographic index in 2019. The purple line was an adaptive association fitted with adaptive Loess regression based on all data points. ASDR, age-standardized disability-adjusted life year rate; AAPC, average annual percentage change; SDI, socio-demographic index; GBD, Global Burden of Disease Study.
